# Zinc in eye health, retinal biology and disease

**DOI:** 10.1016/j.preteyeres.2025.101404

**Published:** 2025-09-19

**Authors:** Brian S. McKay, Andreas M. Grabrucker, Richard B. Thompson, Emily Y. Chew, Imre Lengyel, Héctor González-Iglesias

**Affiliations:** a Department of Ophthalmology and Vision Science and Department of Physiology, The University of Arizona, Tucson, AZ, USA; b Department of Biological Sciences, Bernal Institute, Health Research Institute (HRI), University of Limerick, Limerick, Ireland; c Department of Biochemistry and Molecular Biology, University of Maryland School of Medicine, Baltimore, MD, 21201, USA; d Division of Epidemiology and Clinical Applications, National Eye Institute, National Institutes of Health, Bethesda, MD, USA; e The Wellcome-Wolfson Institute for Experimental Medicine, School of Medicine Dentistry and Biomedical Sciences, Queen’s University Belfast, UK; f Department of Technology and Biotechnology, Dairy Research Institute – Spanish National Research Council (IPLA-CSIC), Spain

**Keywords:** Zinc, Age-related macular degeneration, Drusen, Complement factor H, Retinal pigment epithelium, Retina, Bruch’s membrane, Choroid, Endothelium

## Abstract

Zinc is an essential trace mineral that plays a crucial role in numerous bodily functions, including immune response, wound healing, and protein synthesis. Regarding eye health, zinc is particularly important due to its high concentration, functional abundance, and critical roles in the retina/RPE/choroid complex, where both deficiency and excess can lead to cellular dysfunction. This mineral contributes significantly to the maintenance of the structure and function of the tissues, and it is believed to help protect against oxidative stress, which can damage cells in the eye. The retinal pigment epithelium/choroid complex (RPE/choroid) contains the highest zinc concentration. Therefore, it is unsurprising that several eye disorders associated with this interface are associated with reduced zinc accumulation, and zinc supplementation has become an essential secondary preventive therapy for diseases like age-related macular degeneration (AMD). Despite zinc’s importance in health and diseases of the outer retina, it still needs to be fully understood how zinc participates in cellular and molecular events and how zinc supplementation might be beneficial. However, it appears that adequate zinc levels are essential for retinal health and overall vision, particularly as we age. This review is focused on summarising our current understanding of the biology of zinc, with particular attention paid to the RPE/choroid interface.

## 1. Introduction

Grahn et al. (2001) and Karcioglu (1982) highlighted that knowledge of zinc metabolism in the eye remains incomplete and poorly understood. Since this early work, there has been new research on zinc biology in the retina, including molecular characterization of the zinc transporters and zinc metalloproteins summarised in previous reviews (Barzegar-Befroei et al., 2009; Chappell and Redenti, 2001; Redenti and Chappell, 2005; Ugarte and Osborne, 2001). Despite extensive research, Karcioglu’s statement remains accurate today. The study of zinc biology of the retina has gained momentum following the publication of population-based studies looking at the role of nutrition in preventing age-related macular degeneration (AMD), the leading cause of blindness in high-income countries that is also widely found globally. A potential association between dietary zinc and clinical eye manifestations had intrigued investigators ealy on. Still, the findings of the AREDS group (“A Randomized, Placebo-Controlled, Clinical Trial of High-Dose Supplementation with Vitamins C and E, Beta Carotene, and Zinc for Age-Related Macular Degeneration and Vision Loss: AREDS Report No. 8.” 2001), the Blue Mountain Eye Study (Tan et al., 2008), the Beaver Dam Eye Study (VandenLangenberg et al., 1998) and the Rotterdam Eye Study (van Leeuwen et al., 2005) have captured the interest of the public and clinicians, highlighting a vital need for better understanding of the molecular mechanisms involved in the positive effect of zinc supplementation. Other factors behind the intensified interest in the zinc biology of the eye are our increasing understanding of the functions of zinc in the brain, particularly in association with Alzheimer’s disease (Sensi et al., 2009), as well as a better understanding of zinc transporters (Lichten and Cousins, 2009) and buffering in cells (Colvin et al., 2010). Zinc is an essential cofactor for hundreds of enzymes and, consequently, scores of cellular/tissue functions. From the complicated absorption of zinc from dietary sources to the uptake and transport of zinc throughout the body, the deposition of this cation is tightly controlled. In this review, we look at how zinc is or may be involved in different retinal diseases and summarize our current knowledge of zinc biology in the eye.

## 2. Zinc, eye health and age-related macular degeneration

### 2.1. Zinc deficiency and overload in the eye

#### 2.1.1. Zinc deficiency

Zinc deficiency has been implicated in various ocular pathologies, often presenting with subtle clinical manifestations that can be mis-attributed to other eye conditions (Grahn et al., 2001). These ocular alterations are typically associated with decreased serum zinc levels and, in some cases, reduced tissue zinc concentrations, both of which tend to improve following zinc supplementation (Grahn et al., 2001). Systemic disorders leading to zinc deficiency provide key insights into its ocular effects. Acrodermatitis enteropathica, a rare genetic disorder characterised by impaired intestinal zinc absorption, exemplifies this link. Ocular abnormalities in affected individuals include blepharitis, photophobia, conjunctivitis, corneal opacities, cataracts, superficial punctate opacities, subepithelial nebulous opacities, and linear corneal erosions (Cameron and McClain, 1986; Grahn et al., 2001; Matta et al., 1975; Prabriputaloong and Prakitrittranon, 1992; Racz et al., 1979; Wirsching L., 1962). Gene expression studies in acrodermatitis enteropathica indicate that mutations affect the mRNA of ZIP4, a zinc transport protein, resulting in decreased dietary zinc absorption (Muga and Grider, 1999; Schmitt et al., 2009). Other systemic conditions associated with zinc deficiency include Crohn’s disease, where decreased retinal zinc has been implicated in bilateral maculopathy simulating ‘cherry-red spot’ with vision impairment, which is responsive to zinc supplementation (McClain et al., 1983; Yassur et al., 1981). Thalassemia patients treated with desferrioxamine exhibit altered zinc metabolism, marked by increased faecal and decreased granulocyte zinc levels (De Virgiliis et al., 1988). Long-term total parenteral nutrition has also been associated with impaired visual function and reduced plasma zinc (Vinton et al., 1990). Moreover, abnormal dark adaptation and reduced scotopic retinal responses are common in zinc-deficient states related to alcoholism and hepatic cirrhosis, often necessitating combined zinc and vitamin A supplementation (Grahn et al., 2001; Keeling et al., 1982; Morrison et al., 1978; Russell, 1980, 1982; Russell et al., 1983).

Animal studies corroborate these findings, also emphasising zinc’s role in ocular development. Zinc-deficient diets during rat gestation result in optic cup invagination failure, colobomata, retinal dysplasia, and occasionally anophthalmia (Grahn et al., 2001; Rogers and Hurley, 1987). Marginal zinc and taurine deficiencies during gestation and early postnatal life cause retinal dysplasia and diminished electroretino-graphic responses, though postnatal zinc deficiency alone does not induce retinal morphological changes despite functional impairments (Gottschall-Pass et al., 1997, 1998; Grahn et al., 2001). Severe post-weaning zinc deficiency leads to photoreceptor outer segment degeneration and osmiophilic inclusion bodies in the retinal pigment epithelium (RPE), as demonstrated via electron microscopy (Grahn et al., 2001; Leure-duPree, 1981; Leure-Dupree and Bridges, 1982; Leure-duPree and McClain, 1982).

#### 2.1.2. Zinc overload

While zinc toxicity is rare, excessive exposure can induce ocular toxicity, underscoring its dual role as an essential nutrient and a potential neurotoxin (Plum et al., 2010). *In vitro* studies with isolated rabbit retinas exposed to high zinc concentrations (500 μM or more) have demonstrated neurotoxic effects, including releasing neurotransmitters such as GABA and NMDA. These effects can be mitigated by zinc chelation with diethyldithiocarbamate (Ugarte and Osborne, 2001). Mechanistically, elevated zinc levels activate intracellular pathways that can culminate in neuronal damage. Key processes include generating reactive oxygen species (ROS), disrupting mitochondrial energy production, and inhibiting sodium/potassium-ATPase activity (Ugarte and Osborne, 2001). High zinc concentrations also impair antioxidant defence mechanisms by inactivating enzymes like glutathione peroxidase and glutathione reductase (Splittgerber and Tappel, 1979). Furthermore, zinc can alter the conformation of neurotrophins (Ross et al., 1997) and the survival of retinal cells (Vecino et al., 1998).

### 2.2. Zinc and age-related macular degeneration

The role of zinc in AMD is central to the interest of researchers evaluating the use of zinc supplements for therapy and their adverse effects. The relationship of zinc with AMD was initially studied by Dr. David Newsome, who hypothesized that since zinc deficiency is common in older adults and since zinc is so highly concentrated in the retinal pigment epithelium, zinc supplementation may be a therapy for AMD (Newsome et al., 1988). He conducted a single-centre, randonmize-drandomized, placebo-controlled clinical trial of oral zinc sulfate (80 mg) in 151 patients with AMD in the 1980s. After 12–24 months of follow-up, there was a reduction in visual acuity loss in the zinc-treated group compared with the placebo group. Although the authors empashized that this was a pilot study, and stated that it was “definitely premature to recommend the widespread use of oral zinc, since, as has been pointed out, zinc ingestion can be associated with several potentially serious side effects and complications, including anaemia and worsening of cardiovascular diseases,” this work led to commercially available supplements which were widely publicised and available.

#### 2.2.1. Age-related eye disease study (AREDS)

One of the goals of the National Eye Institute/National Institutes of Health in the 1990s was to study the natural history of two common chronic eye disorders, age-related cataracts and AMD. NEI supported clinical studies that began as an 11-centre study known as the Age-Related Eye Disease Study (AREDS) to evaluate the natural course and prognosis of patients with varying severity of AMD, from no AMD, early, intermediate, and late AMD in one eye. AREDS then pivoted to a randomised controlled clinical trial of oral micronutrients starting in 1992. Other fields of medicine, including oncological and cardiovascular researchers, were testing the role of high doses of antioxidant vitamins such as C, E, and beta-carotene in reducing cardiovascular and cancer risks. While these antioxidant vitamins became part of the AREDS supplements, zinc supplementation was added to this formula because of the potential public health impact of many patients taking zinc, with limited data on its efficacy and adverse effects from a small single-centre study. The dose chosen to be evaluated was 80 mg of zinc oxide (with 2 mg of cupric oxide to offset the potential for copper-deficient anaemia), as this was initially tested. If the AREDS supplements deviated from the dose used in the Newsome study, any negative results would suggest that the researchers did not replicate the initial experiment appropriately.

The AREDS investigators enrolled (1992–2001) 3640 participants with AMD. They were randomised in a factorial design to zinc and copper, the vitamins or the combination of zinc/copper and the vitamins, or placebo from 1992 to 2001. The AREDS results showed that the supplement consisting of the combination of zinc (80 mg zinc oxide), copper (2 mg as cupric oxide) and the combination of vitamins C (500 mg), E (400 international units), and beta-carotene (15 mg) was the most effective of the 4 arms of treatment for reducing the risk of progression to late AMD by 25 % over 5 years (odds ratio [OR], 0.72; 99 % confidence interval [CI], 0.52–0.98) (“A randomised, placebo-controlled, clinical trial of high-dose supplementation with vitamins C and E, beta carotene, and zinc for age-related macular degeneration and vision loss: AREDS report no. 8.,” 2001). Visual acuity loss was also statistically significantly reduced by the combination (OR, 0.73; 99 % CI, 0.54–0.99) and by the zinc alone. No beneficial effect was seen in age-related cataracts.

At enrollment, the study participants were informed of the potential side effects of zinc, including anaemia, decreased high-density lipoprotein cholesterol, and upset stomach. Annual sampling of the blood in a subset of participants showed no increased risk of copper-deficient anaemia or change in serum lipids in those randomised to zinc compared with those not randomised to zinc (Age-Related Eye Disease Study Research Group, 2002). Genitourinary hospitalisations, including conditions such as unspecified urinary tract infection and prostatic hyperplasia in men and stress incontinence in women, were more frequent among participants randomised to the zinc arms (7.5 % vs 4.9 %; P = 0.001 for men and women combined and 8.6 % vs 4.4 %; P = 0.001 for men alone). Hospitalisations for mild/moderate symptoms were also more frequent in participants randomised to zinc arms (9.7 % vs 7.8 %; P = 0.04).

An additional 5 years of epidemiologic follow-up occurred until 2005, when all the participants were provided with AREDS supplements post-clinical trial. In the 10-year follow-up, use of AREDS supplements was associated with a significant (P < 0.001) reduction in the odds of developing advanced AMD or neovascular AMD (odds ratio [OR], 0.66, 95 % confidence interval [CI], 0.53 to 0.83 and OR, 0.60; 95 % CI, 0.47 to 0. 78, respectively). No significant reduction (P < 0.93) was seen for the central geographic atrophy (OR, 1.02; 95 % CI, 0.71 to 1.45). A significant reduction (P < 0.002) in the development of moderate vision loss was seen (OR 0.71; 95 % CI, 0.57 to 0.88). No adverse effects were associated with the AREDS formulation. Mortality was reduced in participants assigned to zinc, especially death from cardiovascular diseases (HR, 0.83; 95 % CI, 0.73 to 0.95; P = 0.008) (Emily Y Chew et al., 2013).

There were concerns that the dosage of zinc may have beeb too high, as the recommended daily allowance is 15 mg. The AREDS participants were also offered Centrum multivitamins in an attempt to standardise any extra vitamins that they may take, and 67 % of all AREDS participants requested Centrum. Centrum also contains 15 mg of zinc, so some participants took as much as 95 mg of zinc. No treatment effect with Centrum was seen in the analyses.

#### 2.2.2. AREDS2

A second randomised controlled clinical trial, AREDS2, was conducted to evaluate the addition of lutein/zeaxanthin and omega-3 polyunsaturated fatty acids to the AREDS supplements (Emily Y. Chew et al., 2013). The secondary aim of this clinical trial was designed to also test a lower level of zinc, 25 mg vs. 80 mg of zinc oxide, in a secondary randomisation. The participants all had at least intermediate AMD in both eyes. This study randomised 4002 participants to a factorial design of lutein/zeaxanthin and omega-3 fatty acids. In the secondary randomisation, 3036 were randomised to high zinc (80 mg) vs. low zinc (25 mg). The results at the 5-year follow-up showed no statistically significant differences between the two doses of zinc (HR: 1.06 [95 % CI: 0.95 to 1.19; P = 0.32]). No clinically or statistically significant differences were observed in reported serious adverse events, including reported gastrointestinal disorders or hospitalisations for genitourinary diseases (Emily Y. Chew et al., 2013) and mortality (Papudesu et al., 2018). The AREDS2 supplement, the result of this second clinical trial, continued with the higher dose of 80 mg of zinc oxide.

#### 2.2.3. Zinc and cognitive impairment

During the course of AREDS, a report emerged in 1994 (Bush et al., 1994) suggesting that increased zinc tested *in vitro* and in rat models resulted in the rapid formation of Alzheimer’s disease-associated beta-amyloid plaques. The AREDS investigators designed a study with a battery of tests, including six validated cognitive tests with 8 components: 1) The Modified Mini-Mental State Examination (3MS); 2) Animal Category; 3) Letter Fluency; 4) Logical Memory Part I and 5) Logical Memory Part II, Wechsler Memory Scale Revised; 6) Immediate Recall and 7) Word List Mean; Buschke Selective Reminding Test; and 8) Digits Backwards. Participants were also tested for depression. Of the 3640 AMD trial participants, 2166 (60 %) completed the AREDS cognitive battery near the end of the clinical trial. These results showed no differences in the cognitive testing (P > 0.5 for all). Therefore, the study did not support a beneficial or harmful effect of antioxidants or zinc and copper on cognition in older adults with AMD (Yaffe et al., 2004).

Cognitive function testing was also conducted in AREDS2. This cognitive study was designed to capture information at baseline and every two years thereafter to test the impact of all the oral supplements on cognitive function. The same battery of tests was administered by certified coordinators by telephone using a standardized protocol. The comparison of the high zinc to low zinc groups again showed no statistically or clinically significant differences in the cognitive function testing with yearly differences of −0.02 (99 % CI: −0.21 to 0.17), P = 0.77 for the composite cognitive function score (Chew et al., 2015). Similarly, the difference between the mini-mental test score (given by telephone as the Telephone Interview Cognitive Status-Modified [TICS-M]) was −0.01 (99 % CI: −0.18 to 0.16, P = 0.89), which was not statistically or clinically significant.

Overall, zinc was found to be clinically significant in treating AMD, especially for reducing the risk of neovascular AMD. In the primary analyses of AREDS, zinc alone or zinc in combination with antioxidant vitamins had a beneficial effect on both the progression to late AMD and vision loss. Side effects may include gastrointestinal upset and increased genitourinary effects. Other adverse effects of anaemia, such as decreased high-density lipoprotein cholesterol and impaired cognitive function, were not found to be statistically or clinically significant within the clinical trials of AREDS and AREDS2. Further consideration may be given to the dose to be administered in the current AREDS2 supplements, given this safety profile and the lack of difference in therapeutic effects between the low and high doses of zinc tested in AREDS2.

## 3. Zinc ion biochemistry in the retina

### 3.1. Zinc ion biochemistry, metabolism and homeostasis

**Fig. 1.**
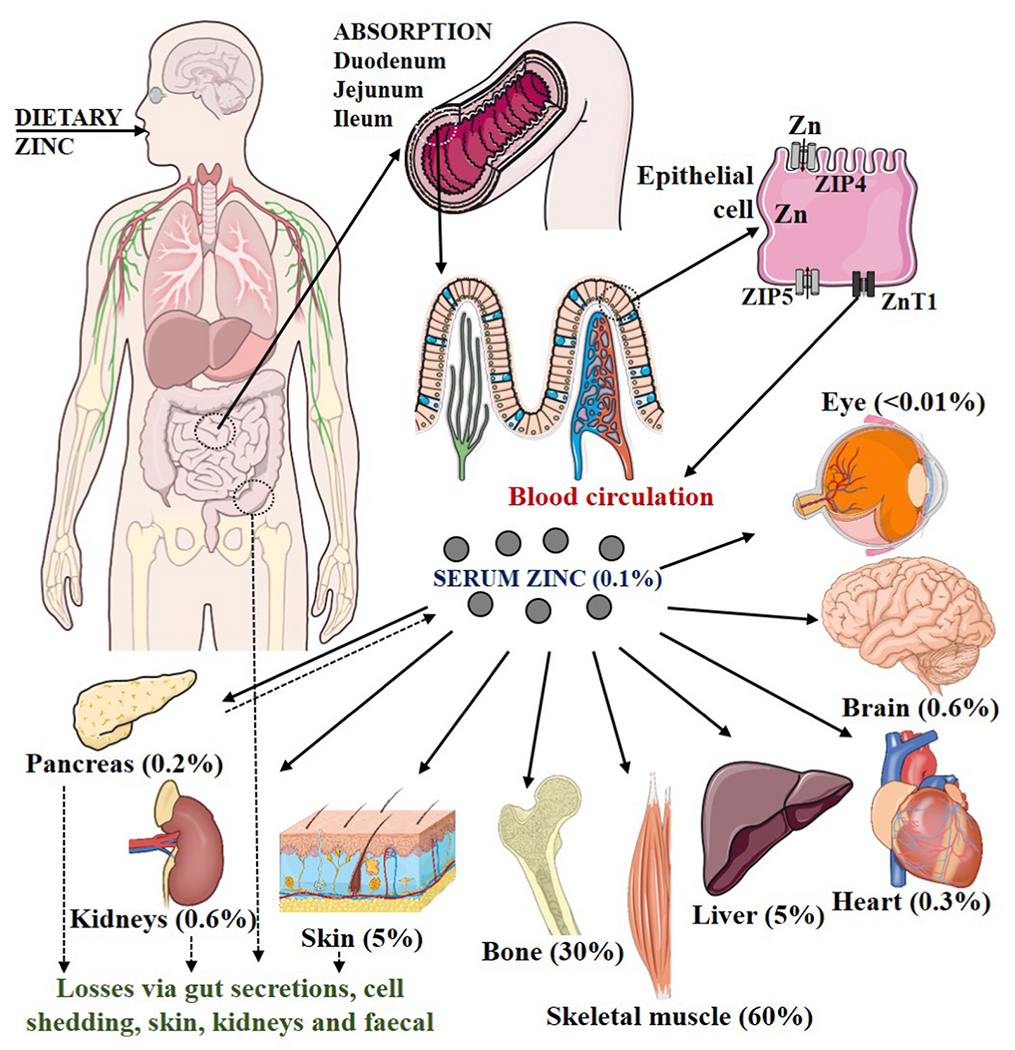
Zinc absorption, distribution, and homeostasis in the human body. Dietary zinc and zinc excreted through pancreatic secretions are absorbed via zinc importer (ZIP4) at the apical surface of the enterocyte and transported into circulation via zinc transporter (ZnT1). In plasma, zinc is primarily bound to albumin (major fraction) and other proteins with a small proportion present in free form and is distributed to peripheral tissues including liver, bone marrow, testis, kidney, skin, heart, skeletal muscle, and pancreas. Zinc homeostasis is maintained by coordinated absorption, distribution, and excretion: losses occur through feces, urine, semen, sweat, cell shedding, skin, and kidneys, with fecal excretion being particularly sensitive to zinc status. During zinc depletion, increased zinc importer-mediated absorption enhances intestinal uptake, while selected tissues (liver or bone) marrow release zinc to maintain plasma zinc levels, whereas zinc is strictly conserved in heart, skeletal muscle, skin, and kidney. Thus, zinc balance is regulated via both the biliary–pancreatic axis during repletion and selective tissue mobilization during deficiency. Images provided by Servier Medical Art (https://smart.servier.com/), licensed under CC BY 4.0 (https://creativecommons.org/licenses/by/4.0/), and by NIAID Visual & Medical Arts (July 10, 2024). NIAID NIH BIOART Source (https://bioart.niaid.nih.gov/).

The adult human body contains approximately 2–3 g of zinc, with the largest fraction localized in skeletal muscle (60–86 %) and bone (20–30 %), followed by skin ~4–8 %) and liver (3–5 %), with smaller amounts in brain, heart, pancreas, eye, and other tissues (Jackson, 1989). Less than 1 % of total zinc is present in plasma, where it is predominantly bound to albumin (~60 %), α2-macroglobulin (~30 %), and transferrin (~10 %), leaving only trace amounts of free zinc (Bloxam et al., 1984; Scott and Bradwell, 1983). This circulating pool, despite its small proportion, is highly dynamic and plays a critical role in distributing zinc throughout the body (Fig. 1).

#### 3.1.1. Dietary zinc absorption and metabolism

Because the human body lacks a specialized zinc storage compartment, a continuous dietary supply is essential to maintain zinc balance (Sangeetha et al., 2022). Zinc requirements vary according to age, sex, and physiological state (e.g., pregnancy, lactation, infancy), and are primarily determined by endogenous losses and dietary bioavailability (Gibson and Ferguson, 1998; Maret and Sandstead, 2006). Current dietary reference intakes (DRIs) for adults are 8 mg/day for women and 11 mg/day for men, with higher requirements during pregnancy (11–12 mg/day) and lactation (12–13 mg/day) (Institute of Medicine US Panel on Micronutrients, 2001).

Zinc metabolism begins with dietary intake, primarily through foods such as red meat, poultry, seafood, whole grains, legumes, and nuts (Trumbo et al., 2001). The bioavailability of zinc is strongly influenced by dietary composition. Zinc from animal-based foods such as red meat, poultry, and shellfish is more efficiently absorbed than zinc from plant-based sources, due to the presence of inhibitors in plant foods, particularly phytates, which chelate zinc and reduce its solubility (Gibson et al., 2006). Diets high in unrefined cereals, legumes, and whole grains can therefore substantially decrease zinc absorption, increasing the risk of deficiency even with apparently adequate intake (Foster and Samman, 2017). Conversely, protein and certain organic acids (e.g., citric acid) can enhance zinc bioavailability (Hamzah et al., 2022).

Zinc homeostasis is primarily maintained through intestinal absorption and endogenous excretion. Absorption occurs along the small intestine, with highest efficiency in the jejunum, while the duodenum, exposed to higher zinc concentrations after meals, also contributes substantially to overall uptake (Lee et al., 1989; Ryu and Aydemir, 2020). The mechanisms of zinc uptake differ between inorganic and organic forms. Inorganic zinc is absorbed as free ions through specific transporters in the small intestine, entering enterocytes via villous epithelium before binding to albumin in the circulation. Organic zinc, complexed with amino acids or peptides, may either be absorbed intact across the intestinal mucosa or hydrolyzed at the brush border, releasing ionic zinc for uptake. Such complexes often bypass inhibitory interactions with dietary constituents, thereby exhibiting higher bioavailability (Maares and Haase, 2020).

Once absorbed into enterocytes, zinc is transported to the circulation, largely bound to albumin, and subsequently delivered to tissues. Endogenous zinc is continuously secreted into the intestinal lumen, from which a portion may be reabsorbed, while the remainder (~0.8–2.7 mg/day in adults) is excreted with feces (Krebs, 2000). This bidirectional process constitutes the major regulatory pathway of systemic zinc balance. During zinc deficiency, fecal zinc losses decrease while intestinal absorption increases, conserving tissue zinc; conversely, under zinc excess, fecal excretion increases without significant changes in absorption (Ryu and Aydemir, 2020).

#### 3.1.2. Zinc compartmentalization, speciation and homeostasis

At the cellular level, zinc is compartmentalized predominantly in the cytoplasm (~50 %), nucleus (30–40 %), and membranes (~10 %). Intracellular zinc exists at very low concentrations as free ions, is sequestered in organelles or vesicles, or tightly bound to metalloproteins and metallothioneins. Collectively, these mechanisms ensure efficient regulation of zinc metabolism, often described as the zinc signal, which contributes to protein structure stabilization, enzymatic activity, and gene regulation (Chen et al., 2024). Zinc ion is well known as a required nutrient that acts as a tightly bound cofactor for hundreds of enzymes (Walsh, 1979), as a central structural element in transcription factors and other DNA-recognizing proteins containing “zinc fingers” (Berg and Shi, 1996; Bertini et al., 2023), and as a signalling ion in the immune system, for example. About 3200 proteins in the human genome are identified as binding zinc. Almost 9 % of all proteins carry zinc, which indicates its importance as a cofactor (Maret, 2013).

Unlike the common monovalent cations, a central concept in considering zinc ions in a biological context is that of speciation: that zinc ion exists in cells and biological fluids in various forms, being bound to other molecules with a broad range of stabilities and functional roles. Thus, zinc ions in biological liquid phases can be found in relatively unstable complexes with water and/or anions such as chloride and hydroxide. These ligands are weakly bound and rapidly exchange with other ligands (Stumm and Morgan, 1996b). Such weakly complexed zinc ion is often characterised as free, “exchangeable,” or “bioavailable” because it diffuses rapidly and is readily available to bind to higher affinity ligands. Exchangeable zinc is maintained in a low, narrow concentration range in most tissues. When intracellular exchangeable zinc levels are high, cells will sequester excess zinc; when zinc levels are low, cells will increase cytosolic zinc influx. Conversely, most zinc ions in biological systems are typically more tightly bound to functional protein domains, such as enzyme active sites, protein folding motifs such as zinc fingers, and storage proteins like metallothioneins (MTs). These sites have multiple ligands belonging to different amino acid residues, often arrayed in the tetrahedral geometry preferred by Zn^2+^, including electronegative atoms such as O, N, and S (Bertini et al., 2023). This illustrates the importance of understanding how the levels of zinc are regulated, particularly how the number and activity of available transporters are regulated.

Thus, in human serum, which ordinarily has a total zinc ion concentration approaching 20 μM, the bulk of the zinc is bound to proteins and amino acids with significant affinities like serum albumin, glutamate, and histidine. Thus, the proportion of “free” zinc is small, on the order of 1 nM or less vs. total in the micromolar range. Therefore, the presence of the storage/multifunction protein MT, reduced glutathione, as well as many metabolic enzymes with zinc active site cofactors (Bertini and Sigel, 2001) ensure that although total cellular zinc ion concentrations may approach 100 μM, measured free zinc levels are only in the range of 1–10 pM (Bozym et al., 2006; Hessels and Merkx, 2015). Since zinc binding is part of the structural folding of many enzymes, and free zinc is so low, proteins likely obtain zinc during synthesis by exchange from MT and zinc-glutathione complexes. In support of this, others have observed small molecule catalysis of zinc addition to the enzyme apocarbonic anhydrase (Pocker and Fong, 1983), which may also occur with other enzymes.

It probably should be stated that zinc ion is in no direct sense an antioxidant, primarily because (unlike transition metals such as iron, copper, or manganese) as a Group IIB(12) element with 3d^10^4s^2^ electronic configuration, it is found only in a single stable ionization form (Zn^2+^) (Cotton et al., 1999) and, consequently, does not participate in redox reactions. While zinc is well known in the classic cytosolic and extracellular Cu, Zn superoxide dismutases (Cu, Zn-SOD (Fridovich, 1995),) it does not participate in the electron transfer reactions and mainly stabilizes the protein structure; it is not required for activity. Zinc evidently can act indirectly to relieve oxidative stress by activating nuclear factor erythroid-2-related factor 2 (Nrf2), which in turn promotes the expression of heme oxygenase, glutathione-s-transferase, and catalase, and production of glutathione, all involved in suppressing oxidative stress (Jarosz et al., 2011; Smith and Loo, 2012; Zhao et al., 2011).

#### 3.1.3. Cellular processes affected by zinc metabolism

Zinc is a vital trace element that influences numerous cellular processes through its structural, catalytic, and regulatory roles. Alterations in zinc metabolism, whether deficiency or excess, can disrupt cellular homeostasis and contribute to disease (see Section 4.1). One of the most fundamental roles of zinc is in enzymatic activity. It serves as a cofactor for hundreds of enzymes involved in DNA replication, RNA transcription, and antioxidant defense. Zinc deficiency impairs enzymes such as SOD, thereby weakening the ability of cells to neutralize ROS (Song et al., 2009). In contrast, excess zinc can interfere with the function of other metal-dependent enzymes by displacing essential cofactors such as iron and copper (Plum et al., 2010). Zinc also plays a critical role in gene expression. Many transcription factors contain zinc finger motifs, which rely on zinc to maintain their structural integrity and DNA-binding capacity. Disrupted zinc levels can alter the expression of genes involved in cell proliferation, differentiation, and apoptosis, leading to developmental abnormalities or uncontrolled cell growth (Dreosti, 2001; Sharif et al., 2012).

In the immune system, zinc is essential for the maturation and function of T cells, B cells, and macrophages (Prasad, 1998, 2008). Deficiency suppresses immune responses and increases susceptibility to infections, while excess zinc may promote inflammation through dysregulated cytokine production (Gruber and Rink, 2013). Zinc functions as a second messenger in cell signaling pathways, influencing calcium signaling, kinase activity, and redox balance. Altered zinc flux can impair signal transduction, thereby affecting processes such as cell migration, adhesion, and stress responses. Furthermore, zinc stabilizes cellular membranes and protects against oxidative damage. Its deficiency heightens oxidative stress, triggering apoptosis and contributing to tissue degeneration (Hübner and Haase, 2021; Kim and Lee, 2021; O’Dell and Browning, 2013). In neurons, zinc imbalance affects synaptic transmission and plasticity, with implications for the development of neurodegenerative diseases (Fan et al., 2024; Vali et al., 2025). Finally, dysregulated zinc transport is increasingly recognized in cancer biology, where altered expression of zinc transporters can promote tumor growth, invasion, and resistance to therapy (Chen et al., 2024). Thus, maintaining proper zinc homeostasis is crucial for cellular health and disease prevention.

### 3.2. Measurements of total zinc

Measurement of total zinc in biological fluids, cells, and tissues is relatively straightforward, except that the methods are typically destructive. Because much of the zinc is tightly bound to proteins, it is usually necessary to extract it with acid, destroying the specimen before it can be analysed by inductively coupled mass spectrometry (ICP-MS), for example. Techniques such as X-ray fluorescence coupled with electron microscopy quantify zinc and other elements with high resolution but tissue destruction (Skoog et al., 2017).

### 3.3. Measurements of free zinc in cells and tissue

Determining the free zinc concentration under various circumstances in tissues, cells, and subcellular compartments is of significant interest. Several molecular tools have been developed to transduce the presence or level of free zinc, mainly as optical changes observed microscopically. The earliest examples were metallochromic indicators because they produced colour changes in solution when they bound zinc: archetypes include zincon, Eriochrome Black T, and dithizone. These were particularly useful for quantifying zinc ions in solution by complexometric titrations but are now obsolete compared with fluorescent (or bioluminescent) zinc indicators since the latter are much more sensitive for use in microscopy.

Fluorescent zinc indicators (often called sensors) exhibit a change in observable fluorescence, such as intensity, colour, lifetime, or polarization upon binding zinc ions. Many such indicators have been described and are available commercially (e.g., FluoZin, FuraZin, TSQ, etc.); typically, they are small organic molecules with an aromatic fluorescent moiety coupled to a metal-binding moiety. For use in biological systems, a key feature is selectivity for zinc ions over other metal ions likely to be present; thus, the classic Ca^2+^ indicators Fura-2 and Indo-1 (Grynkiewicz et al., 1985) actually have a much higher affinity for zinc (and exhibit a similar fluorescence change) compared with calcium. Still, the potential for calcium to interfere with zinc measurements is obvious. At present, the zinc-binding moiety that exhibits the best selectivity for zinc over potential interferents, such as calcium with sub-nanomolar zinc affinity, is the di-(2-picolyl)amine moiety, which is connected to a fluorescein moiety in the classic ZinPyr family of sensors of Burdette and Lippard (Burdette et al., 2001). These indicators are also convenient, reasonably sensitive, and widely used. Most of these indicators exhibit changes in fluorescence intensity (typically increases, e. g., ’turn-on” indicators) upon binding zinc, which helps identify the position of the ion in the microscope but is more problematic for quantification. This is because the measured fluorescence intensity in a microscope or fluorometer is the product of (in simple terms) at least eight factors, according to this Equation (1):

$${\text{I}\;=\;\text{I}}_{\text{ex}}\;\text{x}\;{\text{ε}}_{\lambda }\;\text{x}\;{\text{O}}_{\text{ex}}\;\text{x}\;[\text{flour}]\;\text{x}\;\text{QY}\;\text{x}\;{\text{O}}_{\text{em}}\;\text{x}\;{\text{detect}}_{\text{em}}\text{x}\;\text{Amp}$$

where I_ex_ is the excitation intensity at the excitation wavelength (band), ε_λ_ is the extinction coefficient in that band, O_ex_ is the aggregate efficiency of the excitation optical train, [fluor] is the fluorophore concentration, QY is the fluorophore’s quantum efficiency, O_em_ is the emission optical train’s efficiency at the emission wavelength band, Detect_em_ is the detector efficiency at that band, and Amp is an electronic amplification factor. Of course, these factors also do not consider the presence of interfering fluorophores or the detection of stray excitation light by the detector as fluorescence. Thus, relating a measured fluorescence intensity to a fluorophore quantum yield (and hence zinc ion concentration) is perilous since a variation in any of the factors in Eq. (1) is indistinguishable from a change in zinc concentration. The best practice when using these turn-on indicators is to attempt an *in situ* calibration: the indicator is introduced (for instance) into a cell together with a saturating concentration of zinc using an ionophore such as pyrithione to get the signal level associated with the bound indicator, then a potent, cell-penetrant chelator such as TPEN is added to ensure no zinc is bound to the indicator, such that intensity in between is related to a given fractional saturation of the indicator, and thus free zinc concentration. Under this procedure, most of the factors in Eq. (1) stay the same, and one is left with the apparent change in quantum yield of the indicator associated with the fraction of indicator with zinc bound, and thus free zinc concentration. There are several weaknesses to this widely used two-point calibration procedure. First, ensuring that the measured intensity arises from the indicator alone is difficult. Also, the indicator quantum yield should not change due to binding another species in the cell, like a protein, which may also be zinc-dependent. Finally, we must ensure that the indicator affinity does not change in the buffer or cellular milieu. Because of these intricacies, we suggest intensity/turn-on indicators should be regarded as semi-quantitative measurements.

Tsien (Grynkiewicz et al., 1985) recognized that these issues largely go away if the indicator exhibits a fluorescence spectral change in excitation or emission upon zinc binding. Thus, the indicator shows a spectral shift in excitation or emission upon zinc binding, such that one can relate the ratio of intensities measured at two different emission or excitation wavelengths to the analyte being bound since the wavelength-dependent properties of the indicator are much less susceptible to the issues listed above (ratiometry). Thus, ratiometric indicators beginning with Fura-2 and Indo-1 for calcium ion and pyranine for pH are generally used for their quantification wherever possible.

A continuing limitation of the small molecule zinc indicators is the metal ion binding moiety: the di(2-picolyl) amine moiety has reasonable specificity but limited affinity, and it was clear that sensitivity to picomolar concentrations of free zinc would often be necessary for studies in living systems. We (Fierke and Thompson, 2001) and others (Hessels et al., 2015) concluded that proteins offered zinc-binding sites with high affinity and selectivity that might be useful to engineer sensors. Fierke and Christianson were the first to recognise that subtle changes in the protein amino acid sequence could usefully modulate the affinity, selectivity, and even kinetics of metal ion binding (Thompson and Fierke, 2017). Thus, while human apocarbonic anhydrase II from erythrocytes exhibits high zinc affinity, KD ≈ 10^−12^ M, its association rate constant is extremely slow (10^4^ M^−1^ s^−1^), making its response as a sensor very slow under realistic conditions, reducing usefulness. They also showed that by using a structure-based approach, they could identify modest mutations (e.g., E117A) to “second shell” ligands that bound the metal-ligating histidinyl residues such that the association rate constant increased 800-fold to within an order of magnitude of diffusion-controlled association (Huang et al., 1996).

Several approaches were found for transducing zinc binding to a protein causing a change in fluorescence, particularly as a change in fluorescence intensity ratio at two different wavelengths, ratiometry, or as a change in a lifetime or a change in anisotropy. In particular, the Fierke/Thompson group made use of zinc-dependent binding of fluorescent sulfonamide inhibitors to carbonic anhydrase variants to transduce zinc levels as fluorescence emission ratios at two different excitation wavelengths (Thompson et al., 2002), anisotropy (Thompson et al., 1998) and fluorescence lifetime (Thompson and Patchan, 1995). These protein-based sensors could be introduced inside cells by attaching peptides that promote their importation into cells (Bozym et al., 2006) or expressing them in particular organelles using a suitable vector (McCranor et al., 2012). The Merkx group (Hessels et al., 2015) developed an alternative approach, wherein the binding of Zn^2+^ brings two fluorescent protein moieties comprising a FRET donor-acceptor pair closer together, which is detected by an increase in the acceptor emission accompanied by a concomitant decrease in donor emission. This approach has the advantage of simplicity (compared with the Thompson group’s approach) and ready incorporation by expression into cells; however, the signal change is generally more modest since (being attached) there is always a basal level of FRET between the donor and acceptor moieties. Both these approaches were adapted to bioluminescent transduction, which offers unique advantages (Aper et al., 2016; Thompson et al., 2015).

As a whole, zinc levels are reported in the retina as total and free zinc. Total zinc, usually measured using MS methods, represents the full amount of zinc in a sample. Free zinc is a small portion of the total zinc (<1 %), usually measured using fluorescent-based sensors, which report on zinc that is available for specific, dynamic biological regulatory roles. The bioavailability of zinc is tightly regulated through control of its chemical forms and buffering systems, maintaining exchangeable zinc within physiological limits, typically in the low nanomolar concentrations, to prevent toxicity while supporting the overall enzymatic, structural, and signaling roles for zinc in retinal cells.

## 4. Distribution of zinc in the retina/RPE/choroid complex

**Fig. 2.**
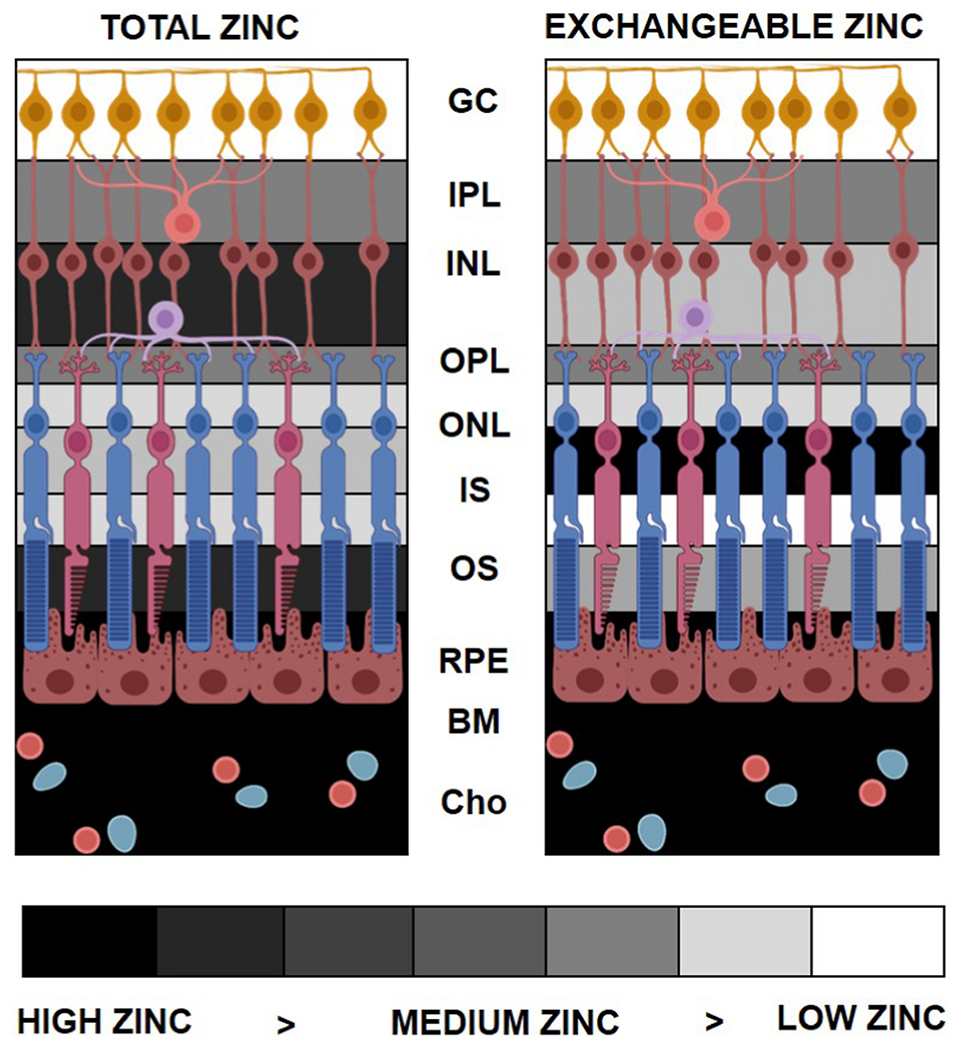
A simplified diagram depicting the cellular organisation of the retina/RPE/choroid complex: Cho, choroid; BM, Bruch’s membrane; RPE, retinal pigment epithelium; OS, outer segment; IS, inner segment; ONL, outer nuclear layer; OPL, outer plexiform layer; INL, inner nuclear layer; IPL, inner plexiform layer and GC, ganglion cell layer. The shaded bar on the right represents exchangeable zinc levels in the different layers based on (Akagi et al., 2001; Kaneda et al., 2005; Ugarte and Osborne, 2001; Wang et al., 2006; Wu et al., 1993). The shaded bar on the left represents total zinc levels based on (Bentley and Grubb, 1991; Bowness et al., 1952; Eckhert, 1983; Erie et al., 2009; Fabe et al., 2000; Galin et al., 1962; Koumantakis et al., 1983; Pålsgård et al., 2001; Rosenthal and Echert, 1980; Tam et al., 1976; Ulshafer et al., 1990; Wills et al., 2008, 2009). The shaded bar on the bottom represents exchangeable zinc levels in the different layers, based on histochemical reactivity. Exchangeable zinc refers to loosely bound, bioavailable zinc that can participate in signaling and enzymatic function, and its distribution reflects changing dynamic cellular processes and functional buffering activity. Created with BioRender.com.

Zinc is the most abundant trace metal in the retina, and its distribution is non-uniform across different retinal layers. In fact, ocular tissue has an unusually high zinc concentration compared to other tissues (Galin et al., 1962), with the highest amount concentrated in the RPE/choroid complex (Grahn et al., 2001; Karcioglu, 1982). A variety of methods have been used for measuring zinc in ocular tissues in different species, including humans (Bentley and Grubb, 1991; Bowness et al., 1952; Eckhert, 1983; Erie et al., 2009; Fabe et al., 2000; Galin et al., 1962; Koumantakis et al., 1983; Pålsgård et al., 2001; Rosenthal and Echert, 1980; Tam et al., 1976; Ulshafer et al., 1990; Wills et al., 2008, 2009). Advanced imaging techniques, such as ICP-MS, have been used to visualise and quantify overall zinc distribution in the retina, providing insights into how zinc is distributed across different retinal layers and how its levels change in disease states (DeToma et al., 2014; Hosseinpour Mashkani et al., 2023; Martínez-García et al., 2023; Rodríguez-Menéndez et al., 2018a; Ugarte et al., 2012). The majority of this zinc is tightly bound to proteins, but a proportion is less tightly bound and thus histochemically reactive (Castro et al., 2017; Wu et al., 2024), as shown by using autometallography (Akagi et al., 2001; Kaneda et al., 2005; Ugarte and Osborne, 2001; X. Wang et al., 2006; Wu et al., 1993), dithizone (Hirayama, 1990) and fluorescent zinc sensors (Lengyel et al., 2007; Lengyel and Onwochei, 2007; Márquez García et al., 2022; Mazzoni et al., 2019; Redenti and Chappell, 2005; Ugarte and Osborne, 1998, 1999). This reactive pool is referred to as “free,” “labile,” “readily releasable,” or “exchangeable” zinc (Colvin et al., 2010; Krall et al., 2021; Maret, 2008). We refer to this pool as exchangeable zinc to highlight its dynamic nature. The distribution of total and exchangeable zinc in the retina/choroid complex is depicted in Fig. 2. The shaded bar on the left represents total zinc levels based on (Bentley and Grubb, 1991; Bowness et al., 1952; Eckhert, 1983; Erie et al., 2009; Fabe et al., 2000; Galin et al., 1962; Koumantakis et al., 1983; Pålsgård et al., 2001; Rosenthal and Echert, 1980; Tam et al., 1976; Ulshafer et al., 1990; Wills et al., 2008, 2009). White represents the lowest and black the highest concentration.

### 4.1. Retina

The most detailed information on exchangeable zinc was obtained using electron microscopy followed by autometallography. Exchangeable zinc has been shown in the Golgi apparatus of ganglion, horizontal, amacrine, and other inner nuclear cells; in the neuronal processes of the outer and inner plexiform layers; and in the nuclei of photoreceptors as wells as Müller cell processes in the outer nuclear layer (Akagi et al., 2001; Kaneda et al., 2005). The Müller cell localization of zinc was also explored in other studies (Lee et al., 2008; Redenti and Chappell, 2004). Neuronal stimulation could induce zinc release (Redenti and Chappell, 2005), providing the first evidence for the neuromodulator role of zinc in the retina (Qian et al., 1997; Williams et al., 2001). It is essential to highlight that tightly bound zinc is also prevalent in this layer, where it plays a pivotal role in forming part of the structure of zinc finger transcription factors, which are essential for gene expression and cellular function (Ugarte and Osborne, 2014).

Zinc also plays a crucial role in the function and regeneration of rhodopsin, a light-sensitive receptor protein found in the rod cells of the retina, affecting vision in low-light conditions. Pålsgård et al. (2001) showed that zinc levels are substantially increased in light-adapted outer segments, indicating the dynamic nature of zinc availability in outer segments. Outer segment zinc labelling was not observed by Akagi et al. (2001). However, zinc has been detected in outer segments by dithizone staining (Hirayama, 1990), autometallography (X. Wang et al., 2006) and TSQ (Ugarte and Osborne, 1998) or zinpyr-1 fluorescence (Lengyel and Onwochei, 2007). Zinc is required to stabilize discs in outer segments (Bettger and O’Dell, 1993), potentially through the stabilization of rhodopsin (Stojanovic et al., 2004; Toledo et al., 2009). In addition, a low-affinity zinc binding to specific histidine sites can lead to destabilisation of rhodopsin (Gleim et al., 2009) and decreased thermal stability (del Valle et al., 2003), suggesting that if zinc is elevated in light conditions, then rhodopsin might be less stable in light than in the dark because of zinc. Instability might be required for the activation of rhodopsin. When rhodopsin absorbs a photon, the 11-cis-retinal component is converted to all-trans-retinal, an isomerisation that triggers a conformational change in the rhodopsin molecule, which leads to its activation (Hargrave, 2001). This zinc-induced destabilisation of rhodopsin might be especially relevant in diseases such as retinitis pigmentosa, which is associated with a Pro23His mutation (Olsson et al., 1992) that causes greater zinc-induced rhodopsin instability (Gleim et al., 2009). Outer segment zinc content can also affect dark adaptation (Oomen, 1974) through the conversion of vitamin A (retinol) to retinal by the zinc-dependent alcohol dehydrogenase (Auld and Bergman, 2008; Kagi and Vallee, 1960), and this activity can be clinically manipulated by zinc supplementation (Morrison et al., 1978). Zinc may also be involved in dark adaptation by affecting the liver’s synthesis or release of retinol-binding proteins (Solomons, 1981). Where and how the reported increase in total zinc in the outer segment (Pålsgård et al., 2001) originates needs further investigation.

In neuronal cells in the retina, zinc also plays a signallng role essential for visual processing and retinal health (Ripps and Chappell, 2014). Zinc is released from neurons from glutamatergic terminals (Paoletti et al., 2009). This release is facilitated by the zinc transporter ZnT3 (see later), which packages zinc into synaptic vehicles (McAllister and Dyck, 2017). Zinc is co-released with glutamate upon neuronal activity, playing a significant role in modulating neurotransmission and synaptic plasticity among photoreceptors, bipolar cells, horizontal cells, amacrine cells, and ganglion cells (Ugarte and Osborne, 2001; Wu et al., 1993). Zinc is an allosteric inhibitor of GluN2A subunit-containing N-methyl-D-aspartate receptors (NMDARs). The inhibitory effect of intracellular Zinc on these receptors is counteracted by zinc export through ZnT1, coupled with GluN2A (Low et al., 2000).

Zinc has a bidirectional effect on α-amino-3-hydroxy-5-methyl-4-isoxazolepropionic acid receptors (AMPARs), often reducing calcium influx and thereby preventing excitotoxicity, and also inhibits γ-aminobutyric acid (GABA)A receptors (GABAARs), regulating horizontal cell GABAergic transmission that contributes to lateral inhibition and contrast sensitivity (Sun et al., 2010). Zinc affects voltage-gated calcium channels (VGCCs) at photoreceptor terminals, reducing calcium entry and thereby modulating glutamate release. Zinc can enter postsynaptic cells through AMPARs, NMDARs, Ca^2+^ channels, and transient receptor potential 7 (TRPM7) channels, and upregulates Kv7 (KCNQ) and large conductance Ca^2+^-activated K^+^ (BK) channels, inhibits TRP1 channels, and either facilitates or inhibits acid-sensing ion channels depending on their subtype. Zinc activates its own metabotropic G-protein coupled receptor (GPR39, mZnR), which is coupled to G-protein alpha q subunit family (Gq) and triggers the release of Ca^2+^ from intracellular stores, leading to downstream signalling through extracellular signal-regulated kinase (ERK), calmodulin kinase II, protein kinase C (PKC), and phosphatidylinositol 3-kinase/AKT (protein kinase B). This receptor also upregulates the potassium (K^+^)/chloride (Cl^−^) cotransporter 2 (KCC2), resulting in a shift in the chloride (Cl^−^) equilibrium potential that promotes GABAAR-mediated inhibition. Intracellular Zinc may also function as a second messenger that activates protein kinases, plays roles in DNA recognition and repair, RNA metabolism, and participates in a number of molecular pathways (for a comprehensive review, see (Benarroch, 2023), and Fig. 2).

Functionally, zinc’s modulation of synaptic activity enhances contrast detection, light adaptation, and signal fidelity in retinal circuits, while also contributing to antioxidant defense and protection from oxidative stress, key factors in retinal degenerative diseases. Dysregulation of zinc homeostasis impairs synaptic function and has been linked to retinitis pigmentosa and AMD (Gilbert et al., 2019). Zinc is released from amacrine cells in the retina, especially following optic nerve injury, which can influence the survival and regeneration of retinal ganglion cells (Li et al., 2017; Liu et al., 2023). Clinical studies indicate that zinc supplementation exerts protective effects in AMD, likely by stabilizing synaptic activity and reducing oxidative stress, underscoring its critical role as a finely tuned regulator of retinal neurotransmission and neuronal health.

### 4.2. Retinal pigment epithelium

The RPE has significantly higher zinc concentrations than other tissues (Samuelson et al., 1993). During periods of systemic zinc deficiency, these levels in the RPE may decrease, but it is resilient to minimal zinc deficiency (Fabe et al., 2000). The pigmentation of the RPE partly regulates zinc content and retention (Kokkinou et al., 2004; Newsome et al., 1992), which is unsurprising given zinc’s role in melanin synthesis and melanosome formation (Furumura et al., 1998). On the other hand, the elevation of extracellular zinc can promote pigmentation and barrier function (Álvarez-Barrios et al., 2023; Pao et al., 2018). The RPE is rich in exchangeable zinc (Akagi et al., 2001; Ugarte and Osborne, 1998; X. Wang et al., 2006), which is localized to the Golgi apparatus (Akagi et al., 2001), melanosomes, and lysosomes (Ugarte and Osborne, 2001; Ulshafer, 1989; Ulshafer et al., 1990; van der Schaft et al., 1992).

Studies have shown that the RPE actively takes up and retains zinc for extended periods (Newsome et al., 1992; Newsome and Rothman, 1987). The RPE in the macula has less zinc than the peripheral retina, and zinc levels in the RPE decrease with age (Tate D. J. et al., 1993). Zinc deficiency in the RPE leads to reduced MT concentration, decreased protein production, and lower activities of enzymes such as catalase, alkaline phosphatase, and alpha-mannosidase. (Tate D. J. et al., 1997, 1995). This deficiency also makes the cells more susceptible to oxidative damage (Miceli et al., 1999). Many of these changes can be remedied by elevating zinc levels in the RPE, which can directly induce transcriptional changes and modulation of key regulatory pathways (Emri et al., 2020).

Zinc plays a dual role in the function of photoreceptors and RPE cells, acting as both a protector and a potential toxin, depending on its concentration and cellular context. Zinc supports enzymatic functions and structural stability in photoreceptors and RPE cells. It contributes to the synthesis of nicotinamide adenine dinucleotide (NAD^+^), a crucial molecule for energy metabolism and cellular survival (Ebeling et al., 2020). Zinc is a key component of the zinc-metallothionein system, which helps neutralize oxidative stress in RPE cells. This system reduces damage caused by ROS, preserving cellular integrity. Moderate zinc supplementation has been shown to induce metallothionein synthesis and reduce oxidative damage in RPE cells, suggesting a therapeutic potential in retinal disorders integrity (Álvarez-Barrios et al., 2021; Rajapakse et al., 2017; Tate D. J. et al., 1999).

Excessive zinc accumulation, especially under conditions of light-induced stress, can lead to photoreceptor and RPE cell death. This toxicity is linked to the depletion of NAD^+^ and ATP, disrupting cellular energy balance. Studies in albino rodents exposed to intense light show that high zinc levels exacerbate retinal degeneration (Sheline et al., 2010). Genetic models with reduced zinc transport or metabolism (e.g., ZnT3-KO, MT3-KO) exhibit less damage, underscoring zinc’s role in retinal vulnerability. Rats fed a low-zinc diet showed reduced retinal damage compared to those on regular or high-zinc diets, indicating that dietary zinc levels can modulate retinal resilience to oxidative stress (Bai et al., 2013).

Zinc also regulate ion channels in the RPE due to its supporting role in photoreceptors, maintaining the visual cycle, and ensuring retinal homeostasis. The RPE relies on tightly controlled ion fluxes, particularly of calcium, potassium, and chloride, to maintain its barrier function and transport nutrients and waste between the retina and choroid. Zinc influences these processes through both direct and indirect modulation of ion channels. In essence, zinc is a key regulator of ion channel dynamics in the RPE, safeguarding retinal health and function (Reichhart and Strauβ, 2020; Wimmers et al., 2007). Zinc can bind to and modulate voltage-gated calcium channels (VGCCs) in RPE cells, thereby reducing calcium influx. This helps regulate intracellular calcium levels, which are crucial for vesicle trafficking, phagocytosis of photoreceptor outer segments, and the secretion of growth factors such as VEGF (Anastassov et al., 2013; Rosenthal et al., 2007). It also affects chloride channels, such as CFTR and CLC family members, which are involved in fluid transport and volume regulation. Zinc may alter channel gating or conductance, influencing RPE hydration and ion balance. Zinc acts as a cofactor for enzymes, such as protein kinases and phosphatases, in the RPE, which regulates the phosphorylation states of ion channels. This can affect channel activity, trafficking, and membrane localization (Wimmers et al., 2007). Zinc also influences oxidative stress responses in the RPE, indirectly preserving ion channel integrity under conditions of retinal stress or aging. Therefore, maintaining proper zinc levels supports the RPE barrier function, photoreceptor survival, and retinal detoxification.

In summary, zinc is vital for the pigmentation of the RPE, influencing melanin synthesis and melanosome formation. Adequate zinc levels help maintain the protective functions of the RPE, while zinc deficiency or dysregulation can compromise these functions, including ion channels, and increase the risk of retinal damage. Given these findings, it was suggested that a lack of zinc might play a role in the development of AMD (Newsome et al., 1988), which led to the inclusion of zinc in the supplement formulation used in the AREDS studies (A randomized, placebo-controlled, clinical trial of high-dose supplementation with vitamins C and E, beta carotene, and zinc for age-related macular degeneration and vision loss: AREDS report no. 8., 2001) (see above). Supplementation with zinc has been shown to slow the progression of AMD, likely through its stabilizing effects on RPE ion channels and antioxidant defense mechanisms.

### 4.3. The Bruch’s membrane

Bruch’s membrane (BM) ha a strategic location between the retina and the choroidal circulation. It is involved in exchanging numerous biomolecules, oxygen, nutrients and waste products between the RPE and choriocapillaris. It plays a crucial role in cell-to-cell communication, cellular differentiation, proliferation, migration and tissue remodelling (Booij et al., 2010a). The BM contains a considerable amount of zinc, especially in the eyes of patients with AMD (Lengyel et al., 2007). As some of this zinc is in the exchangeable form (Lengyel et al., 2007), there may be an extracellular zinc milieu that requires active control for the regulation of extracellular proteins. Proteomic studies have identified a variety of proteins in the BM (Arya et al., 2018; Booij et al., 2010a). These proteins are derived from the RPE and photoreceptors, as well as the choriocapillaris and systemic circulation. Many of these depend on zinc binding to inhibit (Nan et al., 2008) or activate their function to remodel the extracellular matrix (Hussain et al., 2010).

With age, the BM accumulates advanced glycation end-products (AGEs), leading to increased oxidative stress, dysfunction of RPE, structural changes, and lipofuscin accumulation (Glenn et al., 2009). Zinc can mitigate the harmful effects of AGEs as it helps to reduce oxidative stress (Ghodsi and Kheirouri, 2018), lower inflammation (Mohtashamian et al., 2023), and enhance the inhibitory effects of compounds, such as carnosine, against AGE formation (Salahuddin et al., 2014). Zinc can also protect against the glycation of proteins like β-amyloid (Moulahoum et al., 2020).

Proteoglycans (PGs) are heavily glycosylated proteins that act as the “glue” of extracellular matrices, such as BM. PGs have a core protein covalently bonded to glycosaminoglycan (GAG) side chains. Zinc helps stabilize the structure of proteoglycans and GAGs, ensuring they can perform their functions in the BM effectively (Varki et al., 1999).

The interplay between lipids, BM, and zinc is crucial for understanding AMD. Over time, lipids accumulate in BM, contributing to its thickening, which affects nutrient and waste exchange between the retina and the choroidal circulation (Curcio et al., 2011). Zinc regulates lipid metabolism by acting as a cofactor for various enzymes crucial for the synthesis and breakdown of lipids (Olechnowicz et al., 2018). Zinc can help reduce total cholesterol, LDL cholesterol, and triglycerides while potentially increasing HDL cholesterol (Ranasinghe et al., 2015). Due to its antioxidant properties, zinc can help protect lipids from oxidative damage and the formation of harmful lipid peroxides (Olechnowicz et al., 2018).

Overall, understanding the interactions between the components of BM and zinc highlights the importance of maintaining adequate zinc levels to support the function of Bruch’s membrane and prevent retinal diseases.

### 4.4. Choroid

The choroid has the highest zinc concentration in the eye, primarily due to its pigmentation (Wills et al., 2008, 2009). However, relatively little is known about the role and function of zinc in the choroid. Zinc can inhibit tyrosinase activity, thereby affecting melanin production (da Silva et al., 2023). Zinc accumulates in the choroid with age, which could modify inflammatory processes (Gilbert et al., 2019). This accumulation might be linked to changes in zinc metabolism or distribution within the eye as we age (Wills et al., 2008). While some studies suggest that this accumulation could lead to a relative zinc deficiency in other parts of the eye, such as the retina, this idea remains controversial (Erie et al., 2009).

Interestingly, a low zinc diet did not affect the choroidal melanosomes, while the zinc content of the melanosomes in the RPE was decreased (Biesemeier et al., 2012). Zinc has also been shown to affect the fenestration of endothelial cells directly (Cunningham et al., 2021), providing potential mechanisms via zinc that can affect the development and progression of AMD. The exact implications of zinc accumulation in the choroid and its possible contribution to age-related eye conditions are still being explored. Collectively, zinc is unevenly distributed across the retina, RPE, and choroid, with the highest concentrations found in the RPE–choroid complex. It exists in both tightly bound and exchangeable pools, each with distinct functional roles. For example, tightly bound zinc supports enzymatic activity in antioxidant systems such as superoxide dismutase, while exchangeable zinc modulates synaptic transmission in the outer plexiform layer by influencing glutamate release. This spatial and molecular distribution reflects zinc’s diverse contributions to photoreceptor function, antioxidant defense, and retinal signaling.

## 5. Zinc transporters in the retina/RPE/choroid complex

Zinc levels are primarily regulated by 24 transmembrane proteins: 10 zinc efflux transporters (ZnT1-10) and 14 zinc influx transporters (ZIP1-14) (Kambe et al., 2021). These transporters are encoded by two solute-linked carrier (SLC) gene families, SLC30 and SLC39, respectively (Kambe et al., 2015; Liuzzi and Cousins, 2004). Despite the critical role of zinc homeostasis in ocular tissues, the specific localization and function of zinc transporters in the retina and RPE remain largely unknown, both in normal and pathological conditions.

### 5.1. Zinc transporters in the retina

As stated above, localization and roles of zinc transporters in the retina are not fully explored. Previous quantitative RT-PCR data indicates that within the retina, endothelial cells express 6 out of the 10 ZnTs and 12 out of the 14 ZIPs (Barzegar-Befroei et al., 2009)Recent single-cell RNA expression data suggest that all cell types express ZnTs and ZIPs (Yan et al., 2020; Yao et al., 2024; Zuo et al., 2024). However, we know little about whether these genes are transcribed into proteins and what roles those proteins play in health and disease.

Redenti et al. (Redenti and Chappell, 2004) identified ZnT3 protein in several retinal layers, including the inner segments and outer limiting membrane of photoreceptors, the inner nuclear layer, and the outer plexiform layer. The most robust expression was observed in both ganglion and Müller cells, linking ZnT3 and zinc to neuronal transmission (Palmiter et al., 1996). ZnT 1, 3, and 7 were identified in all retina layers by Garcia et al., (Márquez García et al., 2022), with more intensity in layers where there are synaptic connections. Immunolabeling for ZnT7 was also found in optic nerve fibres, ganglion cells, inner and outer plexiform layers, horizontal and amacrine cells, and the photoreceptor outer segments, suggesting that ZnT7 has a broad role in the retina (X. Wang et al., 2006).

### 5.2. Zinc transporters in the RPE

**Fig. 3.**
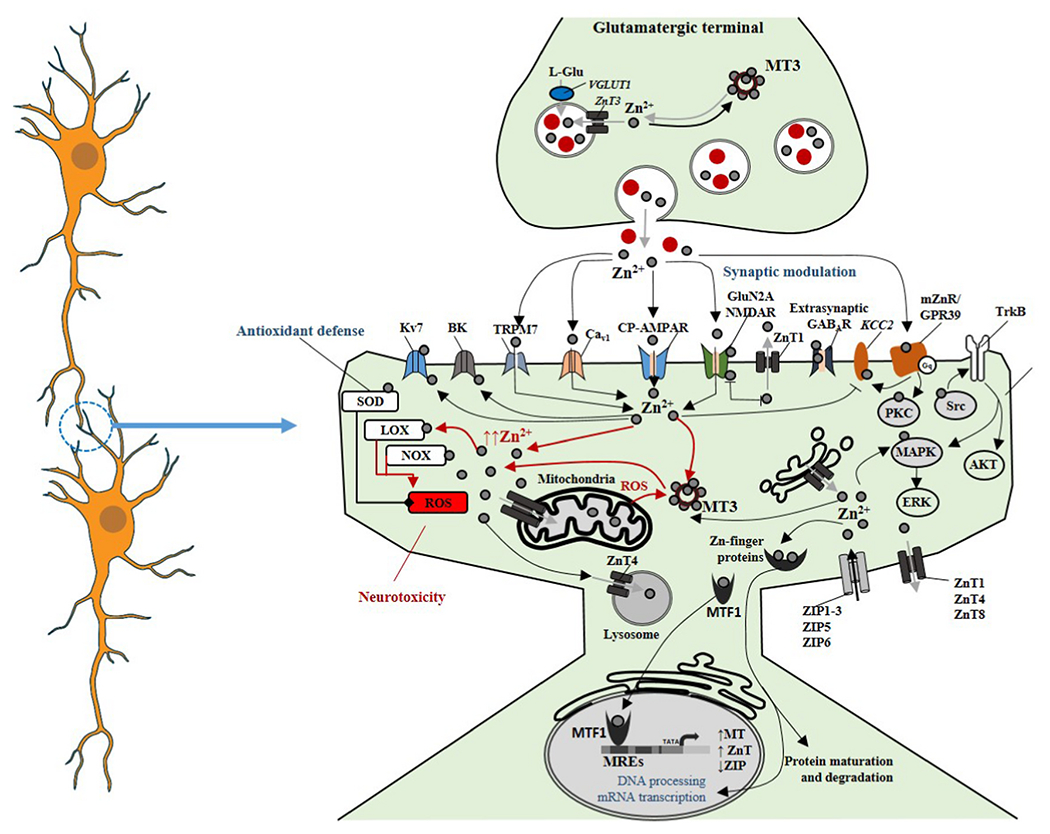
Zinc homeostasis is regulated by zinc transporters of the ZIP (SLC39) and ZnT (SLC30) families and buffered by MTs, especially MT3 (see 8.1 below to further discussion of MTs). Metal-regulatory transcription factor 1 (MTF1), activated by cytosolic Zn ^2+^ , upregulates MT and ZnT genes while repressing ZIP expression. In glutamatergic neurons, zinc is loaded into synaptic vesicles via ZnT3, colocalized with VGLUT1, and modulates neurotransmission by inhibiting GluN2A-containing NMDARs and extrasynaptic GABAARs. Zn enters postsynaptic neurons through Ca ^2+^ Intracellular Zn ^2+^-permeable AMPARs, NMDARs, Cav1 channels, and TRPM7 channels. modulates ion channels (e.g., upregulates Kv7, BK channels) and activates signaling pathways via GPR39, triggering cascades involving ERK, CaMKII, PKC, and AKT. Zn ^2+^ also influences chloride homeostasis through KCC2, promotes neuronal survival via TrkB transactivation, and supports antioxidant defence via SOD activation. Dysregulated Zn levels induce oxidative stress via ROS generation (e.g., NOX, LOX pathways) and are sequestered in mitochondria (via ZIP10) and lysosomes (via ZnT4).

Due to the inclusion of zinc supplements in the AREDS formula and the uptake and retention of zinc in the RPE, zinc regulation has been more extensively studied in these cells. Leung et al. (2008) demonstrated that mRNAs for 21 out of 23 examined zinc transporters were identified in ARPE-19 cells, 20 out of 23 in foetal human RPE, and 16 out of 23 in adult RPE (Leung et al., 2008). ZnT5 was the only transporter not detected in any of the RPE samples analysed. Rezaei et al. (2008) found that apart from ZIP4 and ZIP12, 12 out of the 14 ZIP family transporters were expressed in ARPE-19 cells (Rezaei et al., 2008). Another study reported the expression of 6 out of the 10 ZnTs and 12 out of the 14 ZIPs in ARPE-19 cells, with ZnT2, ZnT3, ZnT8, ZnT10, ZIP2, and ZIP12 not detected (see Fig. 3) (Barzegar-Befroei et al., 2009).

**Fig. 4.**
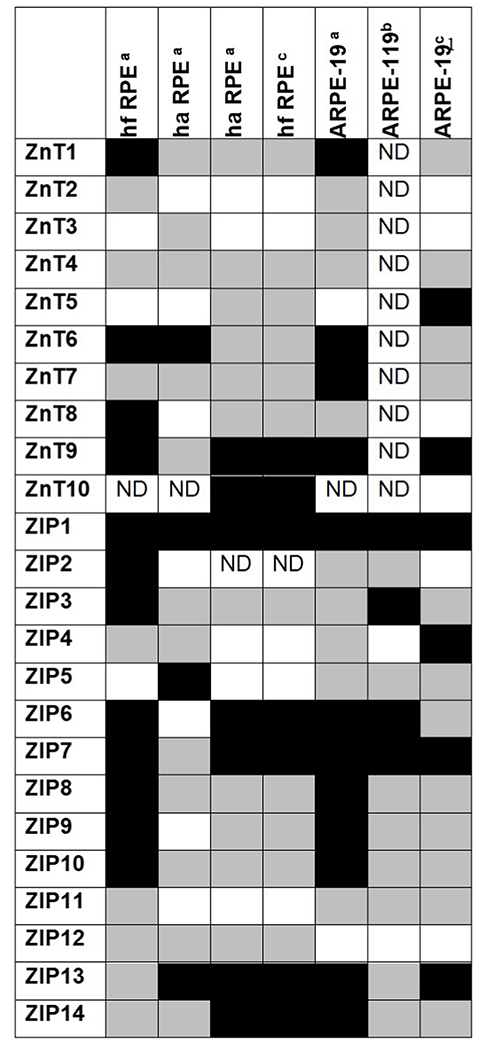
Summary of zinc transporter expression in RPE based on the work of three different laboratories: a (Leung et al., 2008); b (Rezaei et al., 2008); c (Álvarez-Barrios et al., 2023); d (Álvarez-Barrios et al., 2024); d (Barzegar-Befroei et al., 2009). Black represents high; grey, low; and white, with no expression of a gene based on qRT-PCR experiments and RNAseq. Expression levels are compared to selected housekeeping genes. Abbreviations: RPE, retinal pigment epithelium; hf, human fetal; ha human adult; ND, not determined.

We have also measured the expression of all 24 known zinc transporters in ARPE-19 cells (Fig. 4). We found 6 of the 10 ZnTs and 12 of the 14 ZIPs present in ARPE-19 cells. We did not observe expression of ZnT2,3,8 and 10, and ZIP2 and ZIP12 in these cells. Differences between the three laboratories could be attributed to differences in experimental conditions (i.e., batches of cells and/or passage numbers and differentiation state of ARPE-19 cells) or differences in PCR primer design. Still, these data suggest that zinc transporter expression is probably dynamically regulated in RPE cells and may play a role in retina degeneration (Álvarez-Barrios et al., 2023, 2024; Barzegar-Befroei et al., 2009).

#### 5.2.1. Zinc exporters

ZnT transporters typically move zinc from the cytosol to the extracellular space or into intracellular organelles (indicated by grey arrows in Fig. 4). ZnT1, the first plasma membrane zinc transporter discovered (Palmiter and Findley, 1995), is highly expressed in both cultured RPE cells and micro-dissected human RPE (Booij et al., 2010b). This suggests that ZnT1 is likely the primary mediator of zinc efflux in RPE cells. Studies on other cell types (Kirschke and Huang, 2008; Wang et al., 2009) indicate that ZnT1 is present on the apical and basement membranes of RPE cells. Additionally, Leung et al. (2008) demonstrated the perinuclear localization of ZnT1, suggesting that zinc is transported into the nucleus in RPE cells (Leung et al., 2008), probably facilitating transcription.

ZnT2 is an intracellular organelle-associated protein (Lopez and Kelleher, 2009) that typically sequesters zinc into the lysosomes (Kennedy et al., 1994; Wang et al., 2004) and mitochondria (Kelleher and Lopez, 2008). This transporter is thought to play a crucial role in the RPE by supporting the degradation of phagocytosed photoreceptor outer segments, particularly opsins. (Gleim et al., 2009). ZnT2 expression is responsive to environmental changes (Leung et al., 2008; Liuzzi et al., 2001), which may account for the variability in its detection across different studies (Barzegar-Befroei et al., 2009).

While ZnT3 is typically associated with synaptic vesicles (Palmiter et al., 1996), Leung et al. (2008) identified both its expression and translation in RPE cells. They also found that ZnT3 expression is mediated by pigment epithelial-derived factor (Leung et al., 2008).

ZnT4 is associated with intracellular vesicles in the trans-Golgi network (Liuzzi et al., 2001), suggesting its location in vesicles within RPE cells.

ZnT5 expression was not detected in a one report (Leung et al., 2008), but was shown in the RPE in Expressed Sequence Tags (EST) database and another study (Barzegar-Befroei et al., 2009). ZnT5 is involved in vesicular zinc loading and typically localised to the apical cell membrane, although the direction of zinc transport through this transporter is not well understood (Jackson et al., 2007; Kambe et al., 2002; Valentine et al., 2007).

Several studies observed the expression of ZnT6, 7, and 9 (Barzegar-Befroei et al., 2009; Leung et al., 2008). ZnT6 and 7 are involved in the translocation of cytoplasmic zinc into the Golgi network, while ZnT9 is ubiquitously distributed in cells and is thought to be also involved in zinc transport into the nucleus (Sim et al., 2002; Sim and Chow, 1999). As intracellular immunoreactivity of ZnT7 in RPE is very high, transporting zinc into the Golgi might be important for appropriate RPE function.

Although ZnT8 expression was not observed in one experiment (Barzegar-Befroei et al., 2009), it was highly expressed and developmentally regulated in another study (Leung et al., 2008). ZnT8 is typically associated with secretory granules in the pancreas (Chimienti et al., 2004), adrenal gland, thyroid and testis (Chimienti et al., 2004; Murgia et al., 2009), but its potential role in the RPE is not clear.

Like ZnT8, ZnT10 expression was detected by one (Leung et al., 2008), but not another study (Barzegar-Befroei et al., 2009). It is typically localised to the Golgi and plasma membranes, and whether it is involved in RPE function has yet to be identified.

#### 5.2.2. Zinc importers

**Fig. 5.**
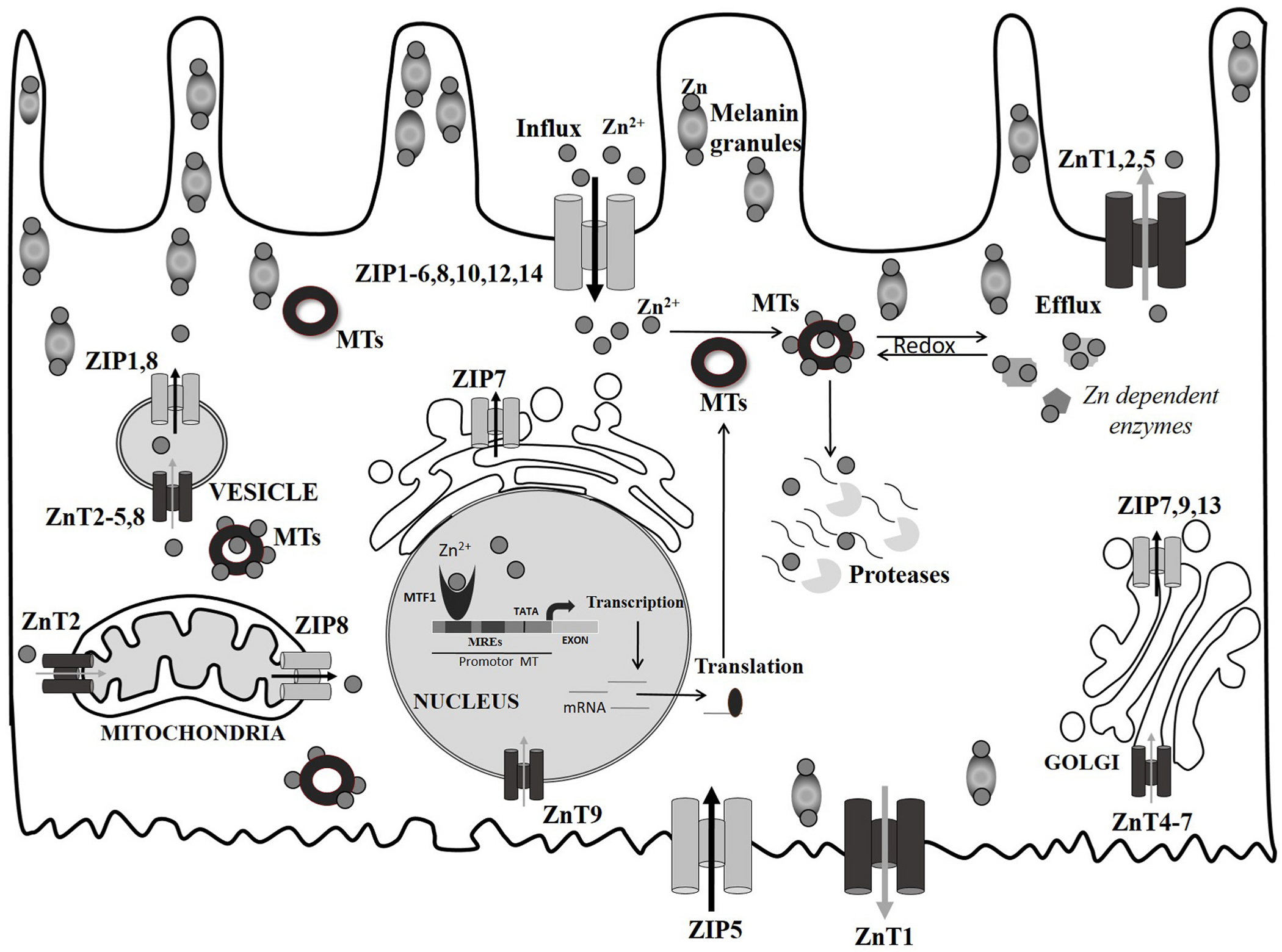
Zinc transporters in RPE. Based on the literature on other cell types and gene expression studies on RPE cells, this figure summarises the understanding of where zinc transporters may be located in RPE cells and includes potential zinc buffering or muffling compartments. For simplicity, endosomes, lysosomes, and secretory vesicles are labelled as “Vesicles” despite their vastly different functions. Arrows represent the direction of zinc transport: grey for ZIPs and dark for ZnTs. ZIP transporters (ZIP1–6, 8, 10, 12, 14) are shown at the plasma membrane, facilitating zinc influx, while ZIP7 is located on the endoplasmic reticulum (ER) and ZIP8 on the mitochondrial membrane. ZnT transporters (ZnT1–5, 8) are depicted on vesicular and mitochondrial membranes, and ZnT4–7 on the Golgi apparatus, mediating zinc efflux or compartmentalization. Metallothioneins are represented as circular structures, act as intracellular zinc buffers. Zinc-dependent enzymes and proteases are also illustrated to highlight functional zinc utilization within the cell. Melanin pigments are represented as black oval structures with zinc circles.

The ZIP family of zinc transporters promotes zinc transport from the extracellular environment or cellular organelles into the cytoplasm (labelled by black arrows in Fig. 5). Relatively little is known about these transporters. ZIP1-6, 8, 12, and 14 are usually located in the plasma membrane and will likely transport zinc into RPE cells from the extracellular space. However, ZIP7 is localised to the ER and Golgi, ZIP8 is present on vesicles and mitochondria, and ZIP13 regulates zinc levels in the Golgi (see summary in (Bafaro et al., 2017; Ehsani et al., 2010)). Although ZIP1 localises to the plasma membrane, it can also shuttle into intracellular vesicles following endocytosis when cells are exposed to excessive zinc. (Belloni-Olivi et al., 2009). Expression of ZIP2 is regulated by extracellular zinc levels outside RPE cells that, in turn, will increase the levels of gamma-glutamylcysteine ligase (a rate-limiting enzyme in glutathione synthesis) and protect against oxidative stress (Ha et al., 2006; Rezaei et al., 2008). The expression of ZIP2 appears to be developmentally regulated and can be altered by neurotropic factors in RPE cells (Leung et al., 2008). ZIP12 is probably the least studied zinc transporter protein. According to the EST database, it is expressed in the retina, kidney, and brain (http://www.ncbi.nlm.nih.gov/nucest). Several studies reported the expression of *SLC39A12* in human fetal and adult RPE but not in ARPE-19 cells (Bafaro et al., 2017; Booij et al., 2010b; Leung et al., 2008; Rezaei et al., 2008).

#### 5.2.3. Eye phenotype in zinc transporter knockout animals

The role of zinc and zinc-dependent mechanisms in development suggests that maintaining proper zinc homeostasis is crucial for normal eye development (Prasad, 1998). Supporting this, studies on transgenic animals have shown that the absence of particular zinc transporter genes can lead to various developmental abnormalities (Dufner-Beattie et al., 2007). For instance, ZIP4 knockout mice exhibit exencephaly, severe growth retardation, hydrocephaly, and unilateral or bilateral anophthalmia. These conditions can be worsened by zinc deficiency and improved with zinc supplementation in heterozygous embryos but not in homozygous embryos (Dufner-Beattie et al., 2007). Additionally, Fukada et al. (2008) reported that ZIP13 is essential for connective tissue development. ZIP13 knockout mice displayed sunken eyes and down-slanting palpebral fissures, which were linked to reduced dermal collagen fibril sizes and thinning of the corneal stroma (Fukada et al., 2008). The absence of ZIP13 also led to improper osteogenesis and craniofacial tissue development. No other animal models are available. Overall, zinc homeostasis in the retina-RPE-choroid complex is orchestrated by a complex network of zinc transporters in the ZnT and ZIP families. These transporters exhibit cell-type-specific expression and subcellular localization, reflecting the nuanced regulation of zinc transport. Disruption of this regulatory system may contribute to retinal pathologies. For example, dysfunction of ZnT3, which transports zinc into synaptic vesicles in photoreceptors and bipolar cells, has been linked to impaired synaptic transmission and increased susceptibility to excitotoxicity, mechanisms implicated in the early stages of retinal degeneration and AMD. This underscores the therapeutic potential of targeting zinc transport mechanisms.

## 6. Metallothioneins and zinc homeostasis in the retina/RPE/choroid complex

MTs belong to a family of cysteine-rich and metal-binding low molecular mass proteins (6–7 kDa) present in all eukaryotes and many prokaryotes (Vasák and Hasler, 2000). MTs consist of two structural domains, the α-domain and the β-domain, with four and three binding sites for divalent metal ions, respectively (Bell and Vallee, 2009). These proteins tightly bind zinc, copper, cadmium, and mercury through the sulfhydryls of cysteine residues, forming metal-thiolate clusters. MT is a multipurpose protein that functions as the main intracellular reservoir of zinc, binding up to seven zinc ions under physiological conditions (Maret, 1995). Zinc buffering mediated by MTs is modulated by the cellular redox state since oxidation of cysteine residues releases zinc and permits its transfer to other metalloproteins, transcription factors, and reservoirs (Kang, 2006).

### 6.1. MTs and cellular zinc homeostasis

The MT family comprises four groups in humans, MT1 to MT4, and includes eleven isoforms and sub isoforms, i.e., MT1A, MT1B, MT1E, MT1F, MT1G, MT1H, MT1M, MT1X, MT2A, MT3, and MT4, encoded by 11 genes located in a cluster on chromosome 16 (Li and Maret, 2008), sharing a high degree of homology both at the nucleotide and amino acid levels (Krezel and Maret, 2021; Vasák, 2005). The cellular functions of MTs include metal ion homeostasis, metal detoxification, antioxidant defence, cellular signalling, immune response, and neuroprotection, among others. Some of these functions are mediated by or require zinc as a cofactor (Bremner, 1987). Cellular zinc homeostasis is tightly controlled by MTs, acting as primary intracellular zinc reservoirs.

MTs bind zinc ions sequentially with graded affinity and exist in metamorphic states, and the metal binding sites may be randomly filled, followed by their rearrangement to a thermodynamically stable state (Krężel and Maret, 2017; Rigby Duncan and Stillman, 2006). Under physiological conditions, zinc binding and release by MTs are mainly conditioned by the cellular redox state, where oxidation of cysteine residues of MTs facilitates the zinc transfer from these proteins to a wide variety of metalloproteins and transcription factors regulating numerous cellular processes (Formigari et al., 2007; Maret, 1995; Zeng et al., 1991). Zinc buffering is dependent on the MT isoform. MT3 binding of zinc is non-cooperative, exhibiting higher metal-binding capacity than ubiquitous MT1 and MT2, while zinc coordination by MT4 results in weaker folding of the protein compared to MT1 (Jacob et al., 1998; Palumaa et al., 2002; Tío et al., 2004; H. Wang et al., 2006).

### 6.2. Distribution of MTs in the retina and RPE/choroid

**Fig. 6.**
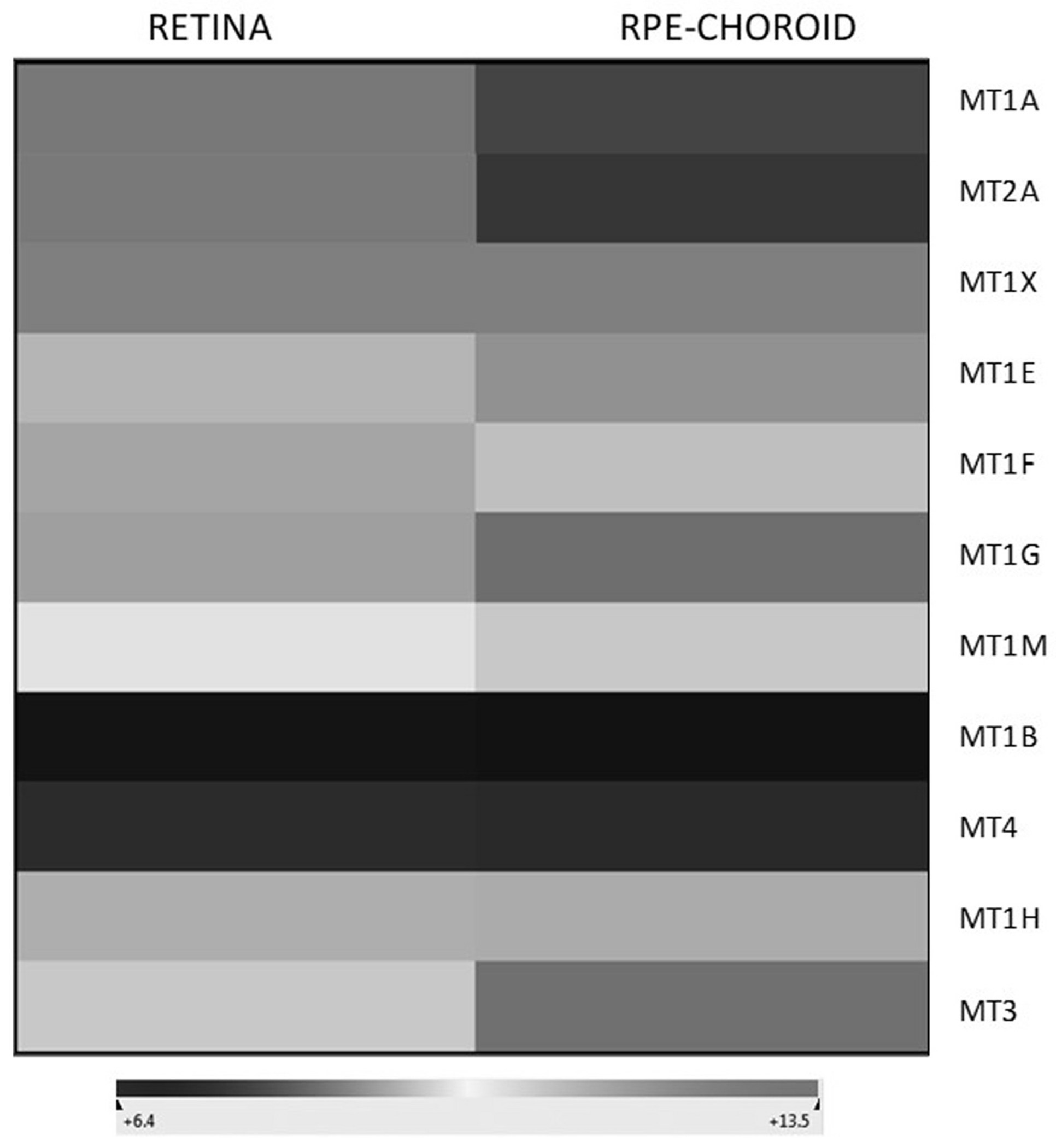
Heat map (whole-expression microarrays) of MT isoforms in the retina (n = 12) and RPE/choroid (n = 8). In arbitrary units, mean values of 6.4–13.5 from biological replicas per tissue are indicated according to the log_2_ scale (Alvarez et al., 2012).

Human eye tissues contain substantial amounts of most MT isoforms according to the gene expression profile of MTs in ocular cells from postmortem donors. Fig. 6 shows the normalized expression levels of MT isoforms restricted to the retina and RPE/choroid. MT1 and MT2 isoforms are expressed to a greater or lesser extent in all of the ocular tissues, being very abundant in the RPE/choroid and retina. At the same time, MT3 expression is restricted mainly to the retina, and the MT4 transcript is not detected (Gonzalez-Iglesias et al., 2014). MT1A, MT2A, and MT1X are the major sub-isoforms, mainly in the RPE/choroid, while MT1G. MT1E, MT1F, and MT1M are expressed at much lower levels (Álvarez-Barrios et al., 2021; Alvarez et al., 2012; Gonzalez-Iglesias et al., 2014).

*In situ* hybridization detected MT isoforms within the RPE and choroids and comparatively less in the sclera, further validated by quantitative PCR also showing MT distribution in the outer nuclear and rods and cones layers (Tate et al., 2002). This specific pattern of MT isoform localization and expression may correlate with tissue-specific activity. For example, the high metabolic oxygen flux and phagocytic activity of the RPE and the high zinc content may require elevated expression levels of MTs for proper function to prevent metal overload, apoptosis, and oxidative stress. The higher expression levels and diversity of MT isoforms in human RPE suggest an MT role in cellular defence against oxidative stress in these ocular tissues that represent natural barriers to external insults (Nakamura et al., 2018).

MT3 is preferentially distributed in the cytoplasm of the retinal ganglion cells and the retinal nerve fiber layer, while being also localized in the cytoplasm of stromal cells in the iris (Gonzalez-Iglesias et al., 2014). The cell-restricted and isoform-specific MT3 detection in retinal ganglion cells may reflect biochemical and/or physiological differences with other MT isoforms since this isoform is mainly localized in neural cells (West et al., 2008). For example, MTs released extracellularly from astrocytes contribute to neuronal survival and regenerative growth of the injured brain (Chung et al., 2008). The damage response mediated by extracellular MTs involves the low-density lipoprotein membrane receptor megalin (LRP2), which binds and internalizes MTs in the neuronal cytoplasm, activating the signal transduction pathways that support neurite outgrowth and survival (Auderset et al., 2016; Hidalgo et al., 2001). The neural retina is part of the central nervous system, therefore MT3 may perform complementary functions within the eye to those previously described.

### 6.3. MT-mediated zinc homeostasis and oxidative stress response in the RPE

The retina and RPE are high energy- and oxygen-consuming tissues directly exposed to sunlight with important metabolic burdens, making them particularly susceptible to oxidative stress (Glickman, 2002). Oxidative stress contributes to the onset and development of many ocular diseases, including AMD (Álvarez-Barrios et al., 2021; Hollyfield et al., 2008; Jarrett and Boulton, 2012). MT expression decreases in the retina-RPE with aging and oxidative stress, which results in the release of zinc within RPE, mainly in the macula (Tate et al., 1993). Müller cells of the fovea from aged retinas showed lower expression levels of specific MT isoforms, including MT3, MT1G, and MT2A, when compared with the same cells of the periphery (Voigt et al., 2021).

Zinc is redox-inert, suggesting an evolutionary conservation of zinc-binding sites in many metalloproteins (Maret, 2000). Zinc complexed with MTs (zinc-MT) acts as a free radical scavenger through the cysteine sulfur ligands along with zinc release, performing the redox chemistry and redox biology of MTs (Chen and Maret, 2001; Gonzalez-Iglesias et al., 2014; Krezel et al., 2007; Krezel and Maret, 2007; Krezel and Maret, 2021; Maret, 2009; Maret and Krezel, 2007; Sutherland et al., 2012; Sutherland and Stillman, 2011). The presence of sulfhydryl groups enables MTs to effectively neutralize superoxide anions and hydroxyl radicals, exhibiting an affinity over 340 times greater than that of reduced glutathione (GSH) (Thornalley and Vasák, 1985). Primary RPE cells cultured in low and normal zinc medium followed by oxidative stress induction showed a decrease in MT content in zinc-deficient cells and an increase in MT levels in adequate zinc RPE cells (Tate et al., 1999). This suggested that zinc protects the thiol groups from oxidative damage and induces *de novo* MT synthesis (Chen et al., 1977; Newsome et al., 1994; Oliver et al., 1992).

Supplementation of primary human RPE cells with different physicochemical forms of zinc, including zinc acetate, zinc chloride, zinc cysteine, and zinc sulfate, increased intracellular MT levels as well as activity of catalase and glutathione peroxidase, mainly with zinc cysteine, protecting these cells from cytotoxic effects induced by H_2_O_2_ and tert-butyl hydroperoxide oxidants (Tate and Newsome, 2006). Recently, *in vitro* culture of retinal ganglion cells transfected with MT1M and those silenced for MT1M demonstrated that up-regulation of MT1M can inhibit RGC cell apoptosis and inflammation and promote RGC cell proliferation through the PI3K/AKT signaling pathway (Li et al., 2020).

ARPE-19 cells contain very high basal levels of MTs, ferritin, and heat shock protein 70 (HSP70), which can be easily up-regulated following specific stimulation with zinc, iron, or heat, respectively (Karlsson et al., 2013), contributing to their great resistance to oxidative stress (Bailey et al., 2004; Kurz et al., 2009; Zareba et al., 2006). Silencing MT gene expression in ARPE-19 cells made them prone to oxidative stress, not reversed by supplementation with 100 μM of zinc (Karlsson and Kurz, 2016). ARPE-19 cells treated with a fragment of mouse liver MT1, the thionein hexapeptide H-Lys-Cys-Thr-Cys-Cys-Ala-OH, protected them against induced oxidative stress and apoptosis (Choudhary et al., 2005). ARPE-19 cells treated with the oxidative stress-inducer paraquat and the aminotetralin agonist 8-hydroxy-2-(di-n-propylamino)-tetralin (8-OH-DPAT) increased the expression of MT1 and other antioxidant enzymes, protecting cells against oxidation (Biswal et al., 2015), as also shown by Ahmed et al. (Ahmed et al., 2016). Similarly, 8-OH-DPAT induced up-regulation of MT1 in mice retinas, protecting the RPE and the neural retina from mitochondrial oxidative stress (Biswal et al., 2015). In addition, MT transfection of the D407 RPE cell line protected against apoptosis and oxidative stress (Lu et al., 2002). Similarly, ARPE-19 cells transfected with MT2A and MT1G genes experienced higher cell survival rates after oxidative damage induced by sodium iodate, while silencing MT2A and MTG genes accelerated cell death after oxidative stress (Wang et al., 2020).

Mechanisms of zinc-MT protection were explored in different eye-derived cell models, including RPE (Alvarez et al., 2012; Gonzalez-Iglesias et al., 2014; González-Iglesias et al., 2017). Oxidative stress was induced in an *in vitro* model of immortalized human RPE cells (HRPEsv) using 2,2′-Azobis(2-amidinopropane) dihydrochloride (AAPH. The effect of zinc treatement on the zinc-MT system was then quantified by mass spectrometry. Zinc at different concentrations (25, 50, or 100 μM ZnSO_4_) specifically induced zinc-MT synthesis (1.6-, 3.6-, and 11.9-fold, respectively), while cells pre-treated with zinc followed by the AAPH oxidative stressor significantly reduced the oxidative stress. Moreover, under steady-state conditions, a Zn_1.4_Cu_0.11_MT stoichiometry was obtained, while under 100 μM zinc pre-treatment and APPH treatment, the MT binding sites were nearly saturated by zinc, i.e., Zn_7.4_Cu_0.03_MT. Conversely, AAPH treatment decreased MT levels (0.4-fold) but increased the number of zinc atoms bound to MTs (Zn_2.07_Cu_0.17_MT). These changes underlie the greater antioxidant capacity of Zn7-MT and the role of the zinc-MT stoichiometry in oxidative stress response by regulating cellular metal homeostasis and transcription factors (Rodríguez-Menéndez et al., 2018a, 2018b).

Links between oxidative stress and zinc-MTs were also discussed in studies with animal models. These were carried out on retinal and RPE homogenates from an early onset macular degeneration monkey model and showed reduced zinc and MT gene expression, along with enzymatic antioxidants (Nicolas et al., 1996). Under a zinc deficient diet, pigmented rats also exhibited reduced levels of MTs in the retina and RPE, as well as increased retinal lipid peroxidation (Miceli et al., 1999). Mice deficient in MT1 and MT2 were subjected to retinal neuron damage by intravitreous N-methyl-D-aspartate (NMDA), affecting cell viability and increasing oxidative stress, thereby increasing retinal ganglion cell death. Conversely, intravitreous injection of zinc in MT-deficient and wild-type mice increased inner retinal MT immunoreactivity. They showed no protection and attenuation of NMDA-induced ganglion cell death, respectively (Suemori et al., 2006). Taken together, these results show that MTs play a central role in intracellular zinc buffering and redox regulation within the retina and RPE. Their isoform-specific expression, such as MT1 and MT2 in glial and RPE cells, and MT3 in neurons, reflects cell-type-specific functions in zinc handling and defense against oxidative stress. Moreover, their dynamic responsiveness to oxidative stress is evidenced by rapid upregulation in response to reactive oxygen species, enabling them to sequester excess zinc and scavenge free radicals. By maintaining zinc homeostasis and limiting oxidative damage, MTs are critical for preserving retinal structure and function.

## 7. Zinc and pigmentary changes

The incidence of AMD is racially biased, occurring in Caucasians 8.1 times more frequently than any other race (Frank et al., 2000; Friedman et al., 1999, 2004). We hypothesized that this was likely related to pigmentation. There is a critical relationship between RPE pigmentation and retinal development. Failure of the RPE to accumulate pigment, as in albinism, causes several retinal defects that result in low vision (Guillery, 1996; Guillery et al., 1975; Sturm et al., 1998). In one form of albinism, ocular albinism (OA), the RPE produces pigment granules that accumulate melanin, but the characteristic retinal manifestations of albinism still occur (Lopez et al., 2008; Schiaffino et al., 1999). OA is not caused by a defect in the enzymes that produce melanin, like other forms of albinism, but rather by a variant cell surface G-protein coupled receptor (GPCR). GPCRs are the most numerous cell surface receptors and communicate extracellular conditions to the cell, typically resulting in a cellular response to external stimuli (Magalhaes et al., 2012; Pavlos and Friedman, 2017). The GPCR in which mutations cause OA is GPR143 (Cortese et al., 2005; Lopez et al., 2008; Schiaffino et al., 1995). We discovered that the ligand for GPR143 is L-DOPA, an intermediate by-product of melanin synthesis (Figueroa et al., 2021; Lopez et al., 2008; McKay, 2019). All pigmented cells in the skin and RPE express GPR143 and release L-DOPA, indicating that GPR143 typically functions in an autocrine loop in pigmented cells.

**Fig. 7.**
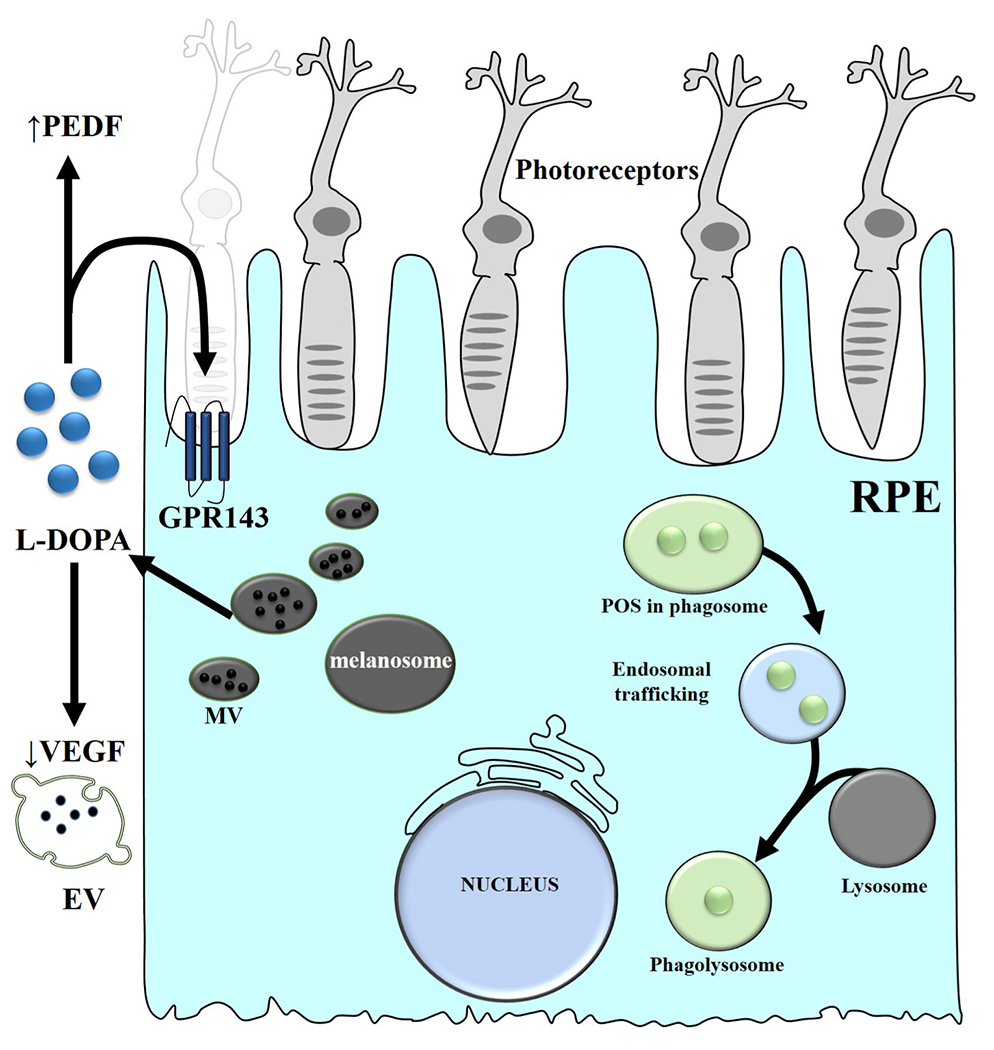
GPR143 functions in two critical endosomal trafficking activities, extracellular vesicles (EV) release and photoreceptor digestion. Activation of GPR143 by L-DOPA halts EV release and upregulates PEDF secretion, both beneficial for retina survival. GPR143 also interacts with melanosomes and microvesicles (MV) to coordinate phtorecepetor outer segment (POS) trafficking and digestion through phagolysosome formation, with zinc contributing to pigment regulation and cellular homeostasis. Mutations in GPR143 cause a complete retinal albinism phenotype despite near normal pigmentation. L-DOPA is produced during melanin synthesis, and loss of the receptor or the ligand leads to the same phenotype with significantly decreased vision.

Several downstream effects of GPR143 signaling directly impact AMD pathogenesis. First, GPR143 signaling upregulates the most potent neurotrophic factor in the eye, pigment epithelium-derived factor (PEDF) (Lopez et al., 2008). In addition to upregulating PEDF release, GPR143 decreases both VEGF and exosome releases (Falk et al., 2012; Locke et al., 2014; Lopez et al., 2008); these activities will decrease the angiogenic potential in the retina and reduce AMD pathogenesis (Fig. 7). Based on these observations, we tested whether L-DOPA and GPR143 signaling would prevent or treat AMD pathology in people. Results in both retrospective analysis and clinical trials indicate that GPR143 signaling prevents and benefits those at risk of vision loss from AMD (Brilliant et al., 2016; Chan et al., 2024; Figueroa et al., 2021; Hyman et al., 2023). The immediate response to GPR143 activation is the cytoplasmic calcium influx. We measured this activity using FURA2 fluorescence by microscopy (Lopez et al., 2008). The cell culture medium used was Hanks’ balanced salt solution, which contained calcium but not zinc. FURA2 binds zinc with higher affinity than Ca and exhibits a similar fluorescence response, but zinc was not included in the assay buffer. Thus, the selectivity, or lack thereof, in divalent ion flux in response to GPR143 activation is unknown. However, the pigment granules have GPR143 on the surface, produce L-DOPA from tyrosinase, and contain a high concentration of zinc (Cunningham et al., 2021; Gilbert et al., 2019; Kokkinou et al., 2005; Lengyel et al., 2007; Newsome et al., 1996; Oliver et al., 1992). It is possible or perhaps likely that GPR143 signaling causes a zinc flux similar to that measured for Ca. Zinc also influences pigmentary dynamics in the RPE through its interaction with melanosomes and endosomal trafficking pathways, including the GPR143 receptor and phagolysosomal processing of photoreceptor outer segments (POS) (Fig. 7). As described in greater detail below, pigment granules have multiple zinc transporters.

This discussion brings together several overlapping ideas that could relate to the mechanism of the zinc effect on AMD incidence. Melanin accumulation in the RPE is similar between various races, suggesting that RPE-specific pigmentation may not underlie the racial bias of AMD incidence (Tung and McKay, 2023; Weiter et al., 1986). Zinc, as discovered by zinc deficiency, does not alter melanin production but does change the pigment granule phenotype and the number of melanin granules observed when selective zinc transporters are knocked out (Álvarez-Barrios et al., 2023; Biesemeier et al., 2012; Cunningham et al., 2021; Emri et al., 2020; Kokkinou et al., 2005; Lengyel et al., 2007; Leung et al., 2012; Wagatsuma et al., 2023). A reduced number or function of pigment granules, where L-DOPA is formed, would negatively impact GPR143 signaling activity. Strong evidence relating GPR143 signaling to AMD incidence and progression has been produced, illustrating that L-DOPA can prevent and treat AMD (Brilliant et al., 2016; Chan et al., 2024; Figueroa et al., 2021; Hyman et al., 2023; Tung and McKay, 2023). This indicates a potential intersection between zinc, pigment granules, and AMD.

RPE is generally thought of as a primary cell type involved in AMD. However, it is unlikely that RPE pigmentation relates to the racial bias of AMD; as discussed above, RPE pigmentation does not vary by race (Weiter et al., 1986). However, just underlying the RPE is the choriocapillaris (choroid), which contains melanocytes (CM) whose pigmentation is racially biased (Weiter et al., 1986). These melanocytes also contain melanin, produce pigment granules, and express GPR143. The racial bias of AMD, as well as the treatment effects of L-DOPA and zinc on AMD, arise perhaps through activity on CM rather than, or in addition to, the RPE. There is currently no reason to expect the activity of zinc transporters to differ between the RPE and CM, and the storage and accumulation of zinc in the pigment granules is likely similar. However, measurements of zinc flux and the comparative expression profiles of RPE and CM zinc transporters are currently lacking.

Further, CM melanin accumulation is similarly race-related, with subjects of African ancestry exhibiting greater melanin levels in CM’s and potentially greater zinc (Kokkinou et al., 2005; Tung and McKay, 2023; Weiter et al., 1986), opening the possibility that zinc may also be reduced in individuals with reduced pigmentation, who are at the greatest risk of developing AMD. Our expectations remain the same: we propose pigmentation protects us from AMD, but the cells responsible are more likely CM rather than RPE cells since AMD incidence varies significantly by race, but RPE pigmentation does not. Also, pigmentation, L-DOPA, and zinc all protect from AMD pathogenesis. Given that the RPE/choroid complex exhibits the most significant accumulation of zinc, has abundant pigment granules, and expresses the receptor for L-DOPA, we suggest that these ‘isolated’ components which protect from AMD are not so isolated and may combine to protect from AMD.

As we consider tissue differences in the melanin granules, one interesting observation is that zinc transporters are cell-type specific, and zinc transporter expression depends on race (Bressler et al., 2008; Emily Y. Chew et al., 2013; Kokkinou et al., 2005; Milton et al., 2005). There are a multitude of zinc transporters, binding proteins, and accessory proteins that have not been evaluated for potential ethnic variation. Given that zinc affects retinal development and the onset of the leading cause of blindness in older adults (AMD), a full study of the population variation in zinc uptake, transport, and accumulation is warranted. Along these lines, while zinc transporter knockouts (KOs) do not cause loss of tyrosinase activity and a lack of melanin formation (Bakker et al., 2022; Leung et al., 2012; Wagatsuma et al., 2023; Wiriyasermkul et al., 2020), the zinc transporter KOs have distinct effects on the storage and development of melanin granules. For example, KO experiments show that ZnT5 and 7 are specifically required for Tryp1 expression and accumulation, not tyrosinase or Tryp2 (Wagatsuma et al., 2022, 2023). Defects in tyrosinase gene (TYR) cause the most notable form of albinism, OCA1, which causes the most significant loss of melanin production and accumulation. Loss of Tryp1 or Tryp2 causes a milder form of albinism, which has reduced but not absent melanin accumulation. Zinc transporter loss results in albinism-like phenotypes reminiscent of ocular cutaneous albinism (OCA) types 3 and 8, along with the characteristic OCA retinal developmental defects (Bakker et al., 2022; Leung et al., 2012; Wagatsuma et al., 2022, 2023; Wiriyasermkul et al., 2020). The downstream OCA phenotype suggests reduced GPR143 signaling due to reduced melanin and L-DOPA production. The reduced accumulation of Tryp1 may be due to a problem with Tryp1 protein folding, as the nascent protein appears to be degraded in the lysosome. An overlapping concept here is that the three related melanogenic enzymes have divalent ions in the active sites; tyrosinase is selective for Cu, but Tryp 2 and 3 may be selective for zinc ions at the active sites (Furumura et al., 1998; Solano, 2018). The activation and transport of these enzymes require cation-specific cation binding at the active site.

## 8. Zinc and the gastrointestinal system-eye axis

### 8.1. Zinc in the gastrointestinal system

The intestine is one of the most regenerative organs in the human body, renewing its epithelium within days (Barker, 2014; Cheng and Leblond, 1974; Sender and Milo, 2021). The processes underlying this renewal depend on the correct sequence and balance of cell proliferation, differentiation, and apoptosis. Intriguingly, these processes require a plethora of zinc-dependent enzymes and signaling proteins. Therefore, zinc status significantly affects intestinal morphology and cell composition, and abnormal zinc status can result in functional alterations.

For example, several animal studies have shown severe consequences of acute and chronic zinc deficiency on the structure and function of the small intestinal epithelium. Based on these studies, zinc seems crucial for maintaining the small intestine mucosal integrity and function (Iwaya et al., 2011; Ranaldi et al., 2013; Rodriguez et al., 1996; Roy et al., 1992; Sturniolo et al., 2002). Zinc deficiency is associated with functional and structural changes in the intestinal epithelium, mucosal necrosis, ulceration, and increased mucosal apoptosis. For example, zinc-deficient rats show a significant reduction of small intestinal length and further morphological changes in the jejunum, including shortening and narrowing of the villi, a decrease in mucosal and crypt cell proliferation, and slower cell migration, leading to a reduction in absorptive surface and renewal capacity of the intestinal epithelium, which were restored by zinc supplementation (Duff and Ettarh, 2002; Elmes, 1977; Koo and Turk, 1977; Southon et al., 1984, 1985; Vignolini et al., 1998). Mechanistically, some of the effects may be caused by zinc-dependent transcription factors that are highly involved in regulating genes necessary for the self-renewal process of the epithelium by intestinal stem cells localized in the base of crypts (Noah et al., 2011; Vela et al., 2015).

Additionally, a study investigating zinc-deficient rats and sheep showed an altered composition of intestinal mucins, likely caused by functional alterations of mucin-secreting goblet cells (Quarterman et al., 1976) that generally produce the protective mucus blanket, which is key to innate host defense. Furthermore, the activity of the zinc-binding matrix metalloproteinase 9 (MMP-9) seems to influence the number of goblet cells and control the secretion of the mucin Muc-2 (Garg et al., 2007).

In addition, zinc has a crucial role in inflammatory bowel disease and prevents increased intestinal permeability (leaky gut). GI inflammation was reported under zinc deficiency status (Hu et al., 2023). Again, zinc supplementation has been shown to benefit mucosal integrity in pathological conditions such as chronic small intestine inflammation (Hu et al., 2023; Kelly et al., 1976; Sturniolo et al., 2002). The functional changes include alterations in the activity of brush border enzymes with low zinc status and inflammatory signaling pathways such as the Nuclear factor kappa B (NF-κB) pathway.

Chronic zinc deficiency also reduces the activity of disaccharidases like sucrase, trehalase, and lactase, as well as leucine aminopeptidase, alkaline phosphatase, and maltase by 30–50 % in the small intestine (Duff and Ettarh, 2002; Tran et al., 2011; Vignolini et al., 1998). These enzymes are essential for the proper digestion of carbohydrates and absorption of saccharides.

In humans, individuals with Acrodermatitis enteropathica, a genetic condition resulting in severe impairment of zinc absorption, show villus atrophy and necrosis in the GI tract (Atherton et al., 1979).

Apart from direct effects on GI cells, zinc status also impacts gut microbiota that, in turn, significantly influence the host organism via zinc-induced modulation of intestinal microflora and their metabolites (Sauer and Grabrucker, 2019; Skalny et al., 2021). For example, acute zinc deficiency in mice resulted in altered microbiota composition. Zinc supplementation was able to rescue the observed changes to the phylum *Proteobacteria* and, more specifically, several bacterial genera such as *Desulfovibrio, Holdemanella, Acetatifactor*, and D*esulfonispora* (Sauer and Grabrucker, 2019). A study in pigs has shown that zinc decreases enterobacteria and clostridial cluster XIV, and increases acetate and butyrate levels (Pieper et al., 2012). Studies in chicks revealed a significant association between zinc status and *Firmicutes*, with the abundance of *Enterobacteriaceae* decreased by higher zinc levels (Nguyen et al., 2021; Reed et al., 2015). In addition, human studies demonstrate that zinc deficiency is associated with reduced gut microbiota biodiversity (Skalny et al., 2021).

Taken together, healthy zinc status has three primary effects on the GI tract: reduction of intestinal damage, anti-inflammatory activity, and modulation of mucosal integrity through gut microbiota composition, among others.

### 8.2. Gut-eye signaling

Gut-to-eye signaling explores the intricate communication between the GI tract and the eyes. This bidirectional communication involves complex networks of neural, metabolic, and immunological signals, mediating a profound interplay between the organs (Campagnoli et al., 2023; Peng et al., 2024).

The enteric nervous system (ENS) and the autonomic nervous system (ANS) facilitate neural connections between the gut and eyes. The Vagus nerve is a conduit for information exchange, influencing ocular functions such as tear production and intraocular pressure (Agassandian et al., 2003; Li et al., 2015; McDougal and Gamlin, 2015). Recent research highlights the role of the gut-eye/brain axis in modulating neuronal signaling, impacting visual processes and ocular health.

Metabolic and hormonal signaling additionally contribute to the communication. Gut-derived peptides, such as ghrelin and cholecystokinin, have been implicated in influencing retinal function and optic nerve health, for example by reducing oxidative damage (Akki et al., 2021). These hormonal messengers traverse the bloodstream, creating a systemic connection between the gut and eyes, with potential implications for conditions like glaucoma and AMD (Gonzalez-Coto et al., 2014; Leite-Moreira et al., 2008; Liu et al., 2014).

Moreover, the composition of the microbiome, exceeding 100 trillion microorganisms, determines interactions with the host organism, regulating immune and metabolic functions relevant to the eye, such as inhibition of pathogens and nutrient metabolism (Napolitano et al., 2021; Scuderi et al., 2022). Dynamic interactions between the gut microbiome and the host’s immune system occur to regulate inflammation (Zysset-Burri et al., 2023). Microbial metabolites profoundly affect the immune system. The gut mucosa houses a vast array of immune cells, and emerging evidence suggests that immune mediators released in response to gut stimuli, such as microbial metabolites, can modulate inflammatory responses within the eyes. Besides, increased intestinal permeability and the potential microbial translocation can contribute to chronic systemic inflammation. Together, this immune communication may have significant implications for AMD.

### 8.3. Gut-eye signaling in AMD

In recent years, the interconnection between gut health and AMD has gained attention, shedding light on the potential role of gut-to-eye signalling in pathogenesis and progression.

Ageing is a genetically determined process that is highly modulated by the environment. The GI tract contributes to the process through changes in the enteric nervous system and alteration of intestinal motility and of the epithelial-mucosal barrier, leading to typical ageing features such as low-grade inflammation (termed “inflammaging”) (Franceschi et al., 2000). Many age-related diseases share an inflammatory pathology, and thus, the gut microbiome has emerged as a critical environmental modifier of aging and age-related diseases.

For example, alterations in the gut microbiome composition, known as dysbiosis, have been associated with inflammatory responses that could contribute to AMD pathogenesis. The gut microbiota’s ability to influence systemic inflammation suggests a potential link between gut dysbiosis and chronic inflammation observed in AMD (Xiao et al., 2023). Increased gut barrier permeability, often observed in conditions associated with gut dysbiosis, can lead to the translocation of microbial products into the systemic circulation, triggering systemic inflammation. In AMD, this systemic inflammation may exacerbate local retinal inflammation and contribute to disease progression (Kugadas et al., 2017). For example, increased translocation of GI metabolites by an increased intestinal permeability may modulate retina-specific immune cells (Andriessen et al., 2016).

Studies indicate that individuals with AMD have increased abundances of the class *Negativicutes*, the genera *Prevotella, Holdemanella, Desulfovibrio*, and *Anaerotruncu*s, as well as the species *Ruminococcus torques* and *Eubacterium ventriosum*, and decreased abundances of the genera *Dorea*, and *Blautia*, and the species *Bacteroides eggerthii* (Lin et al., 2021; Zinkernagel et al., 2017; Zysset-Burri et al., 2020). Seventy-two microbial pathways in subjects with AMD were significantly affected by changes in their microbiome, such as carotenoid biosynthesis, lipid metabolism, fatty acid biosynthesis, and bacterial chemotaxis (Brown et al., 2018).

Moreover, the gut-to-eye axis involves bidirectional communication between the gut and the eyes through neural and hormonal pathways. Dysfunction in these signalling pathways may also impact the regulation of inflammatory processes within the eyes, potentially influencing AMD development. For example, hormonal mediators, such as adipokines and gut-derived peptides, may play a role in gut-to-eye signalling in AMD (Fu et al., 2016). Adipokines, produced by adipose tissue and influenced by gut health, have been linked to retinal inflammation and angiogenesis, processes central to AMD pathophysiology. Gut-derived peptides, including those involved in appetite regulation, could influence retinal function through systemic circulation.

Many of these effects will change the physiology of retinal cells. For example, a recent study showed that mice lacking a GI microbiome have a significantly altered retinal transcriptome compared with mice with intact microbiomes. A total of 396 differentially expressed genes were identified. A pathway analysis based on the differentially expressed set of genes revealed signalling pathways, including insulin-like growth factor (IGF), vascular endothelial growth factor (VEGF), hypoxia-inducible factor (HIF)-1, and 5 AMP-activated protein kinase (Ampk) to be affected (Nadeem et al., 2020).

The AREDS and AREDS2 randomised clinical trials demonstrated that oral supplementation of antioxidant vitamins and minerals, including zinc, can reduce the risk of developing advanced AMD (Chew et al., 2015; SanGiovanni et al., 2007), although the protective mechanisms of these supplements are not entirely understood (Lin et al., 2021). However, it seems likely that the impact of some supplements, such as zinc, on the GI microbiome and GI physiology may be an important underlying mechanism of their beneficial effects. For example, microbiome dysregulation may affect the absorption of some nutrients, leading to a potential deficiency in AMD. In addition, zinc, as a cofactor for SOD, can prevent oxidative stress that contributes to AMD progression not only in the RPE but also the GI epithelium. Zinc may also enhance the effects of other antioxidants in the diet and supplements. Further, zinc may stabilize tight junctions in the GI tract, ensuring proper nutrient transport and waste removal. Besides, AMD involves chronic inflammation and overactivation of the complement system. Zinc helps regulate complement proteins like C3 and C5, reducing inflammatory damage in tissues such as the GI epithelium and retina. Thus, zinc supplements can positively impact GI function by preventing immune activation, aiding tissue renewal, and maintaining the digestive tract’s barrier integrity. For example, a recent study using stem cell-derived GI organoids revealed that zinc signalling in the GI organoid system affects lineage dynamics in gut organogenesis, affects junctional tightness of the intestinal barrier, and promotes pro-inflammatory NF-κB signalling (Sauer et al., 2021). Thus, maintaining adequate zinc status is key in controlling healthy gut-eye signalling.

## 9. Conclusion and perspectives

Zinc plays multiple vital and complex role in retinal physiology. It is essential for visual processing, antioxidant defense, and cellular homeostasis, yet its exchangeable form can be toxic if not tightly regulated. The retina/RPE/choroid complex contains some of the highest zinc concentrations in the body, necessitating robust buffering systems, including MTs, glutathione, melanosomes, and zinc transporters, to limit its exchangeable concentration to physiologically acceptable intracellular levels (Bozym et al., 2006). Cytosolic zinc-binding proteins and small molecules such as glutathione can buffer intracellular zinc by directly binding to it, shuttling it into internal stores, or removing it from the cells altogether (Colvin et al., 2010). The actions of these molecules may hold the key to combating the many eye problems described above. It is well established that MTs are highly but variably expressed in the eye (Tate D. J. et al., 1993; Tate et al., 2002), and they can likely both bind and release zinc during oxidative stress or hypoxia (Maret, 2008). Melanosomes may also serve as zinc buffers and/or stores in the RPE and the choroid (Samuelson et al., 1993; Ulshafer, 1989; Ulshafer et al., 1990). Rhodopsin in the zinc-rich outer segments of photoreceptors may buffer zinc (McCormick, 1985) responding to changes in light levels (Gleim et al., 2009; McCormick, 1985; Tam et al., 1976). Given zinc and other trace metals’ important role in the eye’s degenerative processes, fully understanding the eye’s metallome may be essential in combating eye diseases.

Understanding the intricate relationship between gut health and AMD opens avenues for potential therapeutic interventions. Strategies to modulate the gut microbiota through dietary interventions may offer novel approaches to managing AMD. For example, manipulating the GI microbiota through prebiotics or probiotics is a potential strategy to modulate gut-eye communication and influence ocular health (Scuderi et al., 2022).

Few tissues in the body have total zinc concentrations high enough to release biologically reactive zinc in the range of 20–200 μM. Still, the RPE choroid complex might be one, making this complex a potential target for pharmacological intervention. Zinc buffering-based therapies represent an entirely new approach to pharmacology (Suh et al., 2000). Exchangeable zinc can be selectively removed from the plaques in Alzheimer’s disease by compounds that are relatively weak zinc binders (Bush, 2002). Such compounds are best described as zinc buffers because they do not strip zinc from proteins but merely shift the concentration of exchangeable zinc in the milieu. Given the vital role of zinc in normal visual processing and its presumed involvement in the degeneration of the retina (Ugarte and Osborne, 2001), the goal appears to be the restoration of optimal zinc balance in the eye, which may slow the progression or even prevent the development of AMD.

## Data Availability

No data was used for the research described in the article.
